# A Novel Substrate Radiotracer for Molecular Imaging of SIRT2 Expression and Activity with Positron Emission Tomography

**DOI:** 10.1007/s11307-017-1149-8

**Published:** 2018-08

**Authors:** Robin E. Bonomi, Maxwell Laws, Vadim Popov, Swatabdi Kamal, Shreya Potukutchi, Aleksandr Shavrin, Xin Lu, Nashaat Turkman, Ren-Shyan Liu, Thomas Mangner, Juri G. Gelovani

**Affiliations:** 1 Karmanos Cancer Institute, Wayne State University, Detroit, MI, 48202, USA; 2 PET Center, Wayne State University, Detroit, MI, 48202, USA; 3 National Yang-Ming University School of Medicine, Taipei, Taiwan

**Keywords:** SIRT2, Epigenetic Regulation, F-18, Radiotracer, Positron Emission Tomography, Brain

## Abstract

**Purpose:**

The purpose of this study was to develop a SIRT2-specific substrate-type radiotracer for non-invasive PET imaging of epigenetic regulatory processes mediated by SIRT2 in normal and disease tissues.

**Procedures:**

A library of compounds containing *tert*-butyloxycarbonyl-lysineaminomethylcoumarin backbone was derivatized with fluoroalkyl chains 3–16 carbons in length. SIRT2 most efficiently cleaved the myristoyl, followed by 12-fluorododecanoic and 10fluorodecanoic groups (*K*_cat_/*K*_m_ 716.5 ± 72.8, 615.4 ± 50.5, 269.5 ± 52.1/s mol, respectively). Radiosynthesis of 12- [^18^F]fluorododecanoic aminohexanoicanilide (12-[^18^F]DDAHA) was achieved by nucleophilic radiofluorination of 12-iododecanoic-AHA precursor.

**Results:**

A significantly higher accumulation of 12-[^18^F]DDAHA was observed in MCF-7 and MDA-MB-435 cells *in vitro* as compared to U87, MiaPaCa, and MCF10A, which was consistent with levels of SIRT2 expression. Initial *in vivo* studies using 12-[^18^F]DDAHA conducted in a 9L glioma-bearing rats were discouraging, due to rapid defluorination of this radiotracer upon intravenous administration, as evidenced by significant accumulation of F-18 radioactivity in the skull and other bones, which confounded the interpretation of images of radiotracer accumulation within the tumor and other regions of the brain.

**Conclusions:**

The next generation of SIRT2-specific radiotracers resistant to systemic defluorination should be developed using alternative sites of radiofluorination on the aliphatic chain of DDAHA. A SIRT2-selective radiotracer may provide information about SIRT2 expression and activity in tumors and normal organs and tissues, which may help to better understand the roles of SIRT2 in different diseases.

## Introduction

Over the past two decades, epigenetic regulation has become a rapidly growing, highly innovative and influential field of biology and medicine. One key epigenetic regulatory mechanism involves acetylation and deacetylation of lysine residues of histone core proteins and other critical proteins involved in regulation of gene expression and cell function. Protein acetylation and deacetylation is mediated by histone acetylase transferases (HATs) and histone deacetylases (HDACs), respectively. There are currently 18 HDAC proteins identified in the human genome, which are subdivided into 4 main classes [1]. Classes I, II (a and b), and IV are Zn^2+^-dependent enzymes [2] and include HDACs 1–11, while class III is comprised of seven isoforms of NAD^+^-dependent HDAC enzymes. HDAC class III enzymes are termed the “silent information regulators” or “sirtuins” (SIRTs), originating from the yeast SIR2 homologue, and consist of SIRT1–7 [1]. The SIRT isoforms vary in their cellular localization, with SIRTs 1, 6, and 7 being primarily located in the nucleus, SIRTs 3, 4, and 5 located in mitochondria. SIRT2 is primarily localized in the cytoplasm, but can translocate into the nucleus [3] and deacetylase of cytoplasmic alpha-tubulin and nuclear H4K16 proteins, thereby acting as a mitotic exit and cell cycle regulator [3–8].

Several studies have revealed important roles of SIRT2 in the pathogenesis of various diseases. SIRT2 can play a neuroprotective role in neuorodegenerative diseases, such as Parkinson’s, Alzheimer’s [9], and Huntington’s [10]. SIRT2 has been identified as having both cancer-promoting functions (*i.e*., through stabilization of Myc oncoproteins in breast cancer [11, 12]), as well as cancer-suppressing functions (*i.e*., through tubulin regulation [4]). Therefore, SIRT2 has been identified as a potential drug target for treatment of various neurodegenerative and inflammatory diseases and cancer. However, it is important to assess its activity during pharmacomodulation (*i.e*., activation or inhibition) due to pleiotropic roles of SIRT2 under normal and pathologic conditions. The development of non-invasive molecular imaging approaches for monitoring SIRT2 expression and activity *in vivo* using PET with SIRT2-selective substrate radiotracer may aid in the development of therapies targeting SIRT2.

Understanding structure-activity relationships (SAR) of SIRT2 and its endogenous substrates is important for the development of SIRT2-specific substrate-type radiotracers. The best-studied endogenous substrates of SIRT2 are acetyl-α-tubulin and acetyl-H4K16. SIRT2, similar to other SIRT isoforms namely 4, 5, and 6, is able to cleave larger moieties outside of the acetyl. In particular, it has been demonstrated that SIRT2 can cleave a myristoyl group from the myristoyl(K19 and K20)TNFα [13].

To explore the efficiency with which SIRT2 cleaves larger leaving groups, a focused library of potential substrates was synthesized with *tert*-butyloxycarbonyl-lysine-aminomethylcoumarin (Boc-Lys-AMC) backbone derivatized on the free amino terminus of lysine with a fluoroalkyl chain varying between 3 and 16 carbons in length. Additionally, the influence of fluorine substitution on the ω-carbon in the alkyl chain on substrate affinity and specificity for SIRT2 was evaluated as well. The library of compounds was studied using a fluorogenic enzyme assay with recombinant SIRT2 to determine trends in catalytic efficiency. Furthermore, the SAR of these compounds with SIRT2 was investigated using *in silico* modeling to better understand the observed trends (Fig. S1 Electronic Supplementary Material (ESM)). Based on these studies, the lead compound, the Boc-Lys(12-fluorododecanoyl)-AMC, was identified and its analogue with an aminohexanoicanilide (AHA) backbone was synthesized (12-fluorododecanoicAHA, 12-FDDAHA). The AHA backbone has been used previously to generate effective HDAC class IIa-specific radiotracers: [^18^F]FAHA and [^18^F]TFAHA [14, 15]. Additionally, the radiolabeled analogue of the lead compound, 12-[^18^F]DDAHA, was synthesized from 12-iodododecanoic and 12-bromododecanoic precursors. Finally, the uptake of 12-[^18^F]DDAHA was studied *in vitro* to determine the magnitude of radiotracer accumulation in cells with high *versus* low SIRT2 expression. However, initial *in vivo* studies using 12-[^18^F]DDAHA conducted in a 9L gliomabearing rats were discouraging, due to rapid defluorination of this radiotracer upon intravenous administration, as evidenced by significant accumulation of F-18 radioactivity in the skull and other bones, which confounded the interpretation of images of radiotracer accumulation within the tumor and other regions of the brain. Due to these observations, full characterization of this radiotracer *in vivo* (*i.e*., blocking studies) has not been performed. The next generation of SIRT2-specific radiotracers with reduced systemic defluorination should be developed using alternative sites of radiofluorination on the aliphatic chain of DDAHA (*i.e*., the 3-position).

## Materials and Methods

### Synthesis of ω-Fluorinated Acyl Chains

Details of chemical synthesis and analytical procedures can be found in Electronic Supplementary Materials (ESM) online.

### SIRT Enzyme Assay

Recombinant SIRT1–7 enzymes and BPS1 reference substrate for SIRT2, an oligopeptide corresponding to 379–382 of p53 (Arg-His-Lys-Lys(Ac)-AMC) [16, 17] were purchased from BPS Bioscience (San Diego, CA) and tested using fluorogenic assay. Further details can be found in ESM online.

### Computational Docking Studies

Schrodinger Suite (New York, NY) with the Glide [18] docking program was used for simulating ligand-protein interactions *in silico*. Further details can be found in ESM online.

### Cell Cultures and In Vitro Radiotracer Uptake Studies

The U87MG, MCF-7, MDA-MB-435, and MiaPaca cell lines were used to demonstrate differential levels of 12[^18^F]DDAHA cellular accumulation. Further details can be found in ESM online.

### Intracerebral Glioma Model in Rats

9L tumor cells were implanted into Sprague-Dawley rats and grown *in vivo* for development of a glioma-bearing rat model. Further details can be found in ESM online.

### PET/CT/MR Imaging

PET imaging with 12-[^18^F]DDAHA was conducted in a similar manner as previously published [15]. Details can be found in the ESM online.

### Histology and Immunohistochemistry

After the completion of *in vivo* imaging studies, animals were euthanized under anesthesia and the brain was extracted and prepared for immunohistochemical staining to visualize SIRT2 expression. Details can be found in ESM online.

## Results

**Fig. 1.**
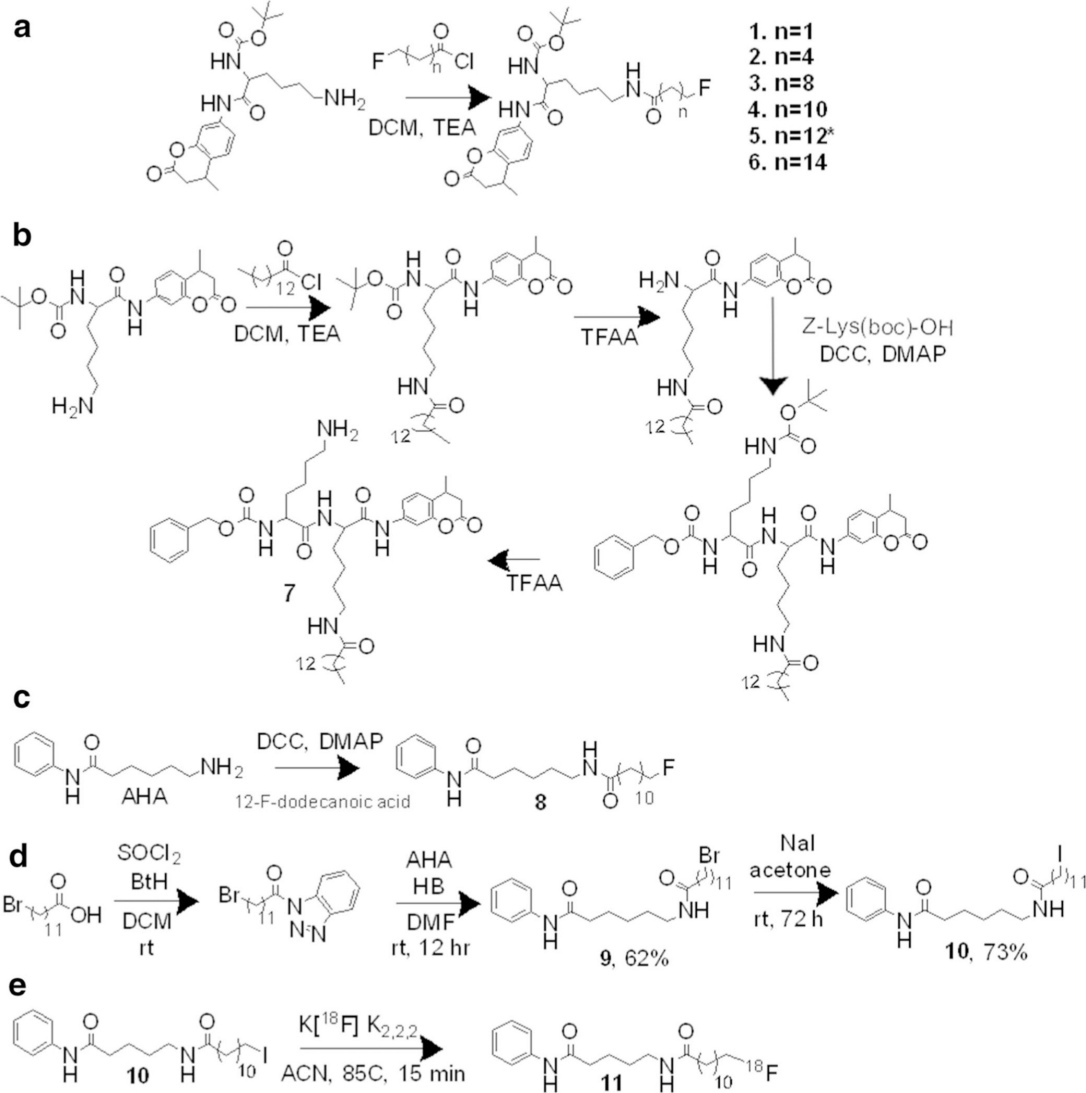
**a** The focused library of compounds synthesized with fluroalkyl chain-derivatized lysines. The backbone used *tert*-butyloxycarbonyl-L-lysine-7-amino-3-methylcoumarin (Boc-Lys-AMC) was coupled to fluoroalkyl chains, varying in length from 3 carbons to 16 carbons using acetyl chloride coupling. **b** To experimentally determine the effect of the cap on the selectivity of a substrate for SIRT2, we developed a di-lysine substrate, with a more peptidic backbone, Cbz-lys-lys(myr)-AMC. This allows for head-to-head comparison between the Boc-lys(myr)-AMC. **c** The synthesis of both the cold (non-radiolabeled) analogue (**9**) and **d** precursors for 12-[^18^F]DDAHA, 12-BrDDAHA (**9**), and 12-IDDAHA (**10**). **e** The 12-[^18^F]DDAHA (11) radiotracer was synthesized from the iodinated precursor and the in dry acetonitrile in 85 °C for 15 min.

The library of compounds with terminally fluorinated acyl chains was synthesized through traditional coupling methods of the fluorinated carboxylic acid chain with the lysine amino derivative *via in-situ* formation of an acetyl chloride in moderate yields of 20–55 %. Synthesis of the fluorinated carboxylic acid chains employed a novel approach: diethylaminosulfur trifluoride was used for non-selective fluorination of the *n*-hydroxycarboxylic acid forming the acetyl fluoride and fluorination of the terminal carbon. Selective hydrolysis of the acetyl fluoride was achieved using flash chromatography over silica gel to form a mono-ω-fluorinated chain due to the abrupt change in pH. The purity of final compounds in the library was 9 95 %, as determined by ^1^H-NMR, ^13^C-NMR, ^19^F-NMR, and HRMS (Fig. 1a).

Compound 7 was synthesized from Cbz-Lys-OH (Bachem) and Boc-Lys-AMC (Bachem), which was derivatized to Boc-Lys(myr)-AMC. Following myristoylation, the Boc protecting group was removed and the resulting free lysine amino terminus was coupled with the Cbz-Lys-OH group. The four-step synthesis yielded 7 in a yield of 12 % with 9 95 % purity as determined by ^1^HNMR, ^13^C-NMR, and HRMS (Fig. 1b).

### Radiosynthesis

12-Fluorododecanoyl-AHA (12-FDDAHA) and 12-[^18^F]dodecanoylAHA (12-[^18^F]DDAHA) were synthesized as non-radiolabeled and radiolabeled analog of **4** (Fig. 1c–e, S1 ESM). The initial radiosynthesis of 12-[^18^F]DDAHA (**11**) was performed using a brominated precursor, 12Br-DDAHA (**9**); however, due to low radiochemical yield, the iodinated precursor, 12I-DDAHA (10), was used for optimization of the radiolabeling procedure and improvement of radiosynthetic yield. The iodinated precursor (**10**) was synthesized from the brominated precursor in a high yield of 73 % using literature procedure [19]. The radiochemical yield achieved with precursor **10** was 5-fold higher than that with precursor **9**. The highest radiofluorination yields were achieved using precursor **10** in acetonitrile at 85–90 °C. 12[^18^F]DDAHA was obtained with purity of > 95 % (Fig. 1d, and S3 ESM) and a decay-corrected radiochemical yield of 12 % (total preparation time 66 min) with specific activity of 259 GBq/mmol.

### Biochemical Characterization

**Fig. 2.**
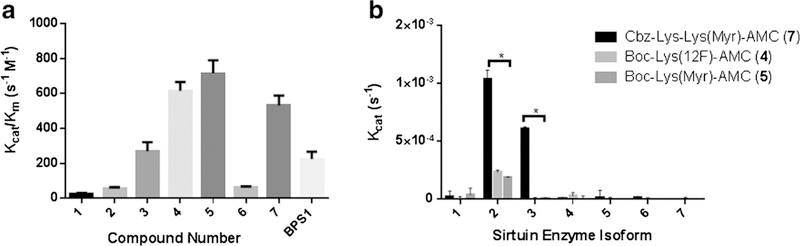
Biochemical assay data from *Fluor de Lys assay*®. **a** The catalytic efficiency (*K*_cat_/*K*_m_) for SIRT2 against different length fluoroalkyl chain leaving groups to assess the structure activity relationship between these compounds and SIRT2. The error bars represent the standard deviation of *N* = 6 experiments. These values were taken from 15-min incubation to capture linear phase kinetics. **b** Compound **7** is not selective for SIRT2 whereas compounds **4** and **5** are selective for SIRT2. These results were taken from 40-min incubation of substrate with enzyme. The error bars represent the standard deviations of the means with significance of *P* < 0.05 denoted by *, as determined by two-way ANOVA. **c** The table details the effect on *K*_m_ (amount of substrate necessary for half of *V*_max_), *V*_max_ (the maximal turnover velocity for a given substrate in SIRT2), and *K*_cat_ (the catalytic efficiency for a given substrate with SIRT2). This data is determined in linear phase kinetics of SIRT2 with 15-min of incubation; therefore, this data is slightly different than the values in Fig. 2b.

Results from *in vitro* characterization of this library of compounds using fluorogenic assay demonstrated a positive correlation between the substrate efficiency for SIRT2 and the increasing length of the leaving alkyl chain. Compound **5** containing the myristoyl group was the most efficient substrate of SIRT2 with *K*_cat_/*K*_m_ = 715.6 ± 72.8/s mol followed by compound **4** (fluorododecanoyl) and compound **3** (fluorodecanoyl) with *K*_cat_/*K*_m_ of 615.4 ± 50.5/s mol and 269.5 ± 52.1/s mol, respectively. In contrast, compound 6 containing the fluoropalmitoyl leaving group exhibited a significant decrease in substrate efficiency for SIRT2 (30.4 ± 7.3/s mol), as compared to compounds containing shorter fluoroalkyl chains. The reference substrate, p53(379–382)K382ac-AMC (**BPS1**) exhibited *K*_cat_/*K*_m_ = 275 ± 42/s mol (Fig. 2, Table 1).

To assess the influence of a “cap” modification on SIRT2 selectivity, compounds **4**, **5**, and **7** were evaluated against a panel of recombinant sirtuins (SIRT1–7). Compound 7 was cleaved efficiently by SIRT2, SIRT3, and, to a lesser extent, by SIRT6. In contrast, **4** and **5** were cleaved efficiently only by SIRT2 (Fig. S2 ESM). To validate the substrate efficiency of **4** with SIRT2, a competitive inhibition assay with AGK2 (SIRT2-specific inhibitor) was performed, which yielded an IC_50_ of 28.21 μmol of AGK2 (Fig. S2 ESM).

### In Silico Modeling

*In silico* modeling studies demonstrated that positioning of compounds **4** and **5** in the SIRT2 active site resulted in a smaller distance between the carbonyl carbon and the nicotinamide-ribose ester linkage (Fig. S4a and S4b ESM). This may contribute to enhanced substrate efficiency of these compounds.

### In Vitro Radiotracer Accumulation Studies

**Fig. 3.**
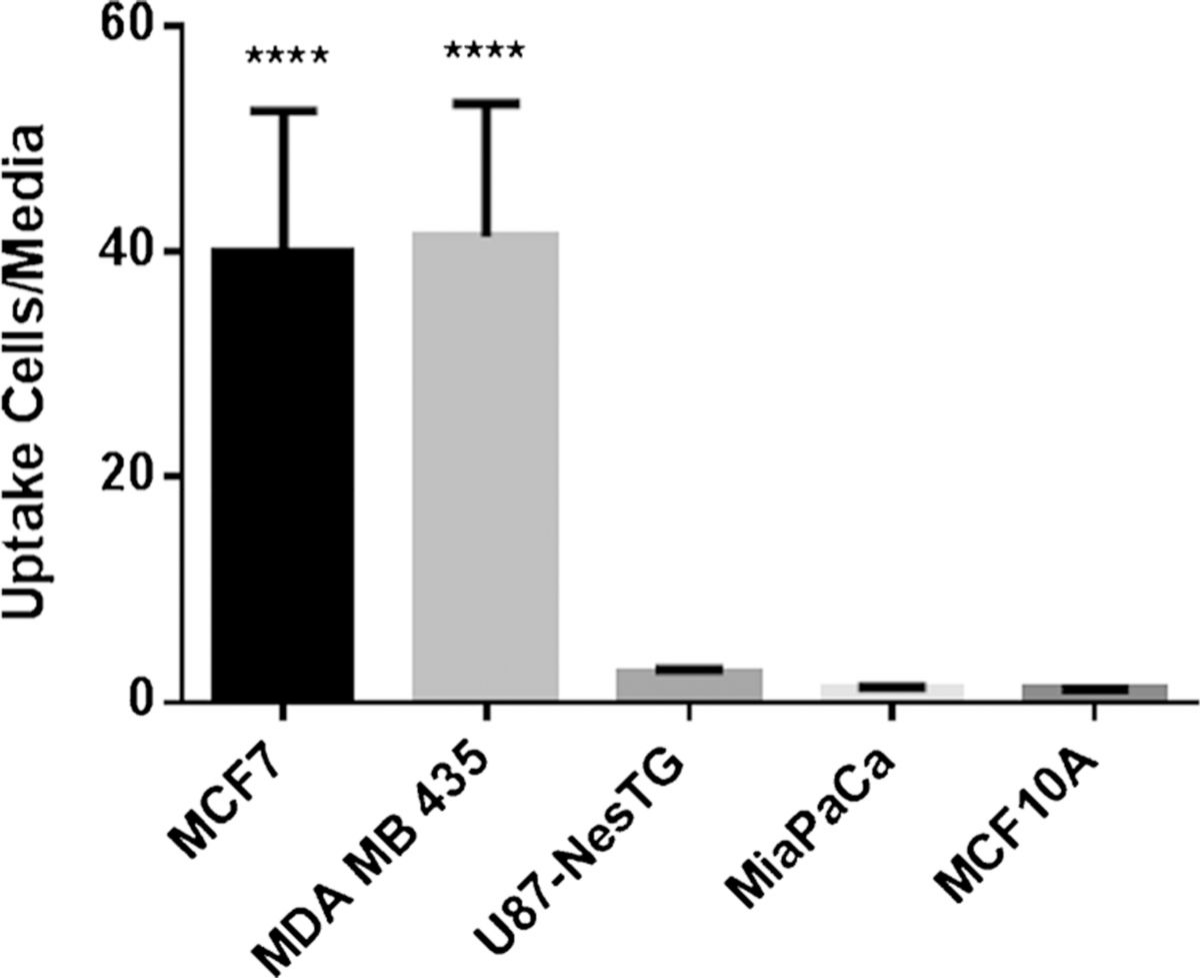
The radiotracer 12-[^18^F]DDAHA (**11**) was used in cellular uptake studies for tumor cell lines. The error bars represent the standard deviation for *N* = 4 for each cell line. Where **** represents *P* < 0.05 as determined by one-way ANOVA and Tukey’s multiple comparisons test, this value is significantly larger than the other three values.

MDA-MB-435 and MCF-7 with high levels of SIRT2 expression [20] demonstrated significantly higher levels of 12-[^18^F]DDAHA accumulation (Fig. 3), as compared to U87MG > MiaPaCa > MCF10A cells, which are known to express lower levels of SIRT2 [20] (very low levels of SIRT2 in U87 reported in Maxwell, et al. 2011 [21]). The extracellular-to-intracellular radioactivity concentration equilibrium was achieved in about 1 min of incubation with 12[^18^F]DDAHA, as the result of high lipophilicity of this radiotracer cLogP = 5.32 which facilitates passive diffusion of intact (parent) compound across the cell membranes in and out of the cells (data not shown). The accumulation and retention of 12-[^18^F]DDAHA-derived radioactivity is due to much higher negative polarity of ADPR-O-[^18^F]dodecanoate, which is the theoretical product of enzymatic reaction of SIRT2 and 12-[^18^F]DDAHA.

### PET/CT/MR Imaging

**Fig. 4.**
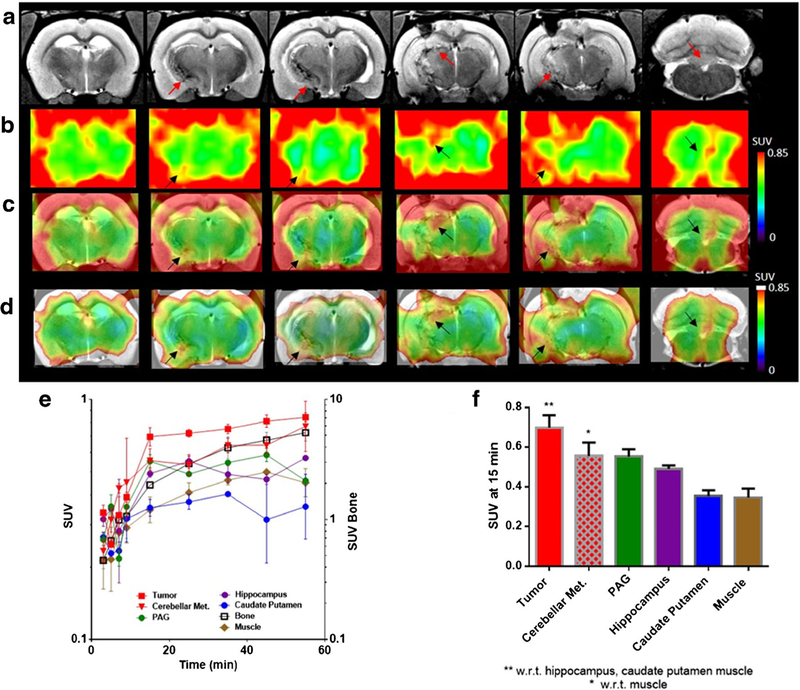
**a** T2-weighted MR image sliced coronally through the 9L glioma lesion and hippocampus with red arrows indicating areas of tumor lesion. **b** Dynamic PET images at 15-min post administration of 12-[^18^F]DDAHA NIH lookup table (LUT). **c** Dynamic PET images post administration of 12-[^18^F]DDAHA overlaid onto T2-weighted MR images using the NIH LUT. **d** The same images as in **c,** but with the NIH + white LUT, to better visualize the internal structures due to the high degree of defluorination occurring, which is seen as F-18 accumulation in the bone causing a “halo” effect around the cortical region due to partial volume effect of bone signal into cortex. **e** Time activity curves for each region of interest within the brain over 60 min of dynamic PET images displayed as log (SUV) *vs* time. The error bars represent standard deviation of the voxel values within each ROI. **f** A visual representation of differential washout between tumor and other regions of the brain at 15-min post i.v. administration of 12-[^18^F]DDAHA, where error bars represent standard deviations and * or ** represent *P* < 0.05 as found from one-way ANOVA.

Dynamic PET/CT/MR imaging of a 9L glioma tumor-bearing rat with [^18^F]-12-DDFAHA demonstrated increased tracer derived-radioactivity accumulation and retention within the tumor *versus* normal brain structures between 15 and 20 min post i.v. administration (Fig. 4). However, interpretation of PET/CT/MR images of [^18^F]-12-DDFAHAderived radioactivity accumulation in 9L gliomas and in the brain cortex was challenging due to high levels of radioactivity accumulation in the adjacent scull bones, which created “spillover” artifacts (Fig. 4b, c). High levels of radioactivity in the bone were caused by rapid defluorination of [^18^F]-12-DDFAHA after i.v. administration. To reduce the appearance of bone radioactivity-causing artifacts in brain PET images, we used the NIH + white (max) lookup table for color coding the range of radioactivity concentrations in the images (Fig. 4d).

### Immunohistochemical Validation of SIRT2 Expression in 9L Glioma and Normal Brain Structures

**Fig. 5.**
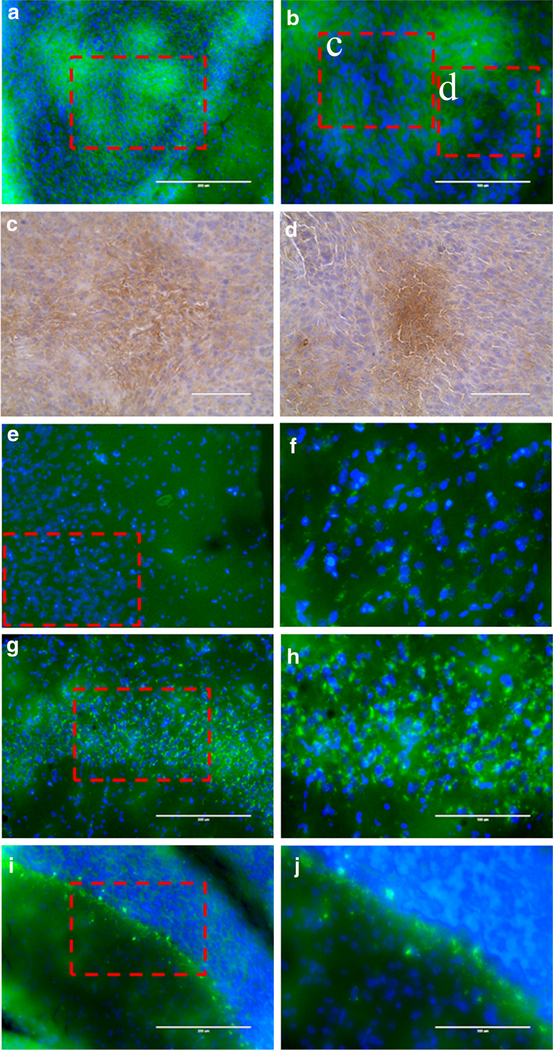
Fluorescence and light microscopy images of different regions of immunostained tissue sections of the rat brain with 9L gliomas obtained at × 200 (**a**, **c**, **e**, **g**, **i**) and × 400 (**b**, **d**, **f**, **h**, **j**). Immunofluorescent images of SIRT2 expression in (**a**, **b**) 9L glioma, (**e**, **f**) contralateral brain cortex, (**g**, **h**) CA2–3 region of contralateral hippocampus, and (**i**, **j**) cerebellum. Immunohistochemically stained adjacent tissue sections of intracerebral 9L gliomas reveal high levels of HIF-1α expression (**c**, **d**) corresponding to regions of decreased SIRT2 expression in panel.

Fluorescence microscopy of sections of rat brains revealed heterogeneously distributed patchy appearing circular-shaped regions of SIRT2 expression in 9L gliomas (Fig. 5a, b), as well as in the peripheral infiltrating zones. Immunihistochemical staining for HIF-1a expression in adjacent tissue sections confirmed that the areas of reduced SIRT2 expression in tumors co-localized with areas of increased HIF-1a expression (Fig. 5c, d). In the cerebral cortex, SIRT2 expression was expressed mainly in the neurons, with the highest concentrations in perinuclear regions of pyramidal neurons and lower levels in the axons (Fig. 5e, f). In the hippocampus, the level of SIRT2 expression was highest in CA2 and CA3 neurons and had a similar sub-cellular perinuclear localization pattern, as in cortical neurons (Fig. 5g, h). In the cerebellum, SIRT2 was strongly overexpressed in Purkinje cells, with perinuclear localization stronger than in cortical and hippocampal neurons; SIRT2 was also observed in neurons in the molecular layer at somewhat lower levels of expression than in Purkinje cells, but was almost entirely absent in the granular layer neurons (Fig. 5i, j).

## Discussion

To develop the first-generation substrate-type radiotracer selective to SIRT2, it was necessary to understand the structure-activity relationships of SIRT2 with various natural and synthetic substrates. Previously, it has been reported that in addition to an acetyl group, SIRT2 can also cleave long acyl chains from lysine residues, including a myristoyl moiety [22]. Other HDAC class III enzymes, such as SIRT3 and SIRT6, have also been reported to cleave myristoylated lysines of the H3K9 peptide [23, 24] and K19–20TNF-α [22]. However, there are no data on the effects of fluorine substitution on the ω-carbon in alkyl chains on different lengths on SIRT2 catalytic efficiency.

Our current studies demonstrate that catalytic efficiency of SIRT2 gradually increases with the elongation of fluoroalkyl chain from 3 to 12 carbons. The catalytic efficiency of SIRT2 for fluorododecanoyl chain (*K*_cat_/*K*_m_ = 615.4 ± 50.5/s mol) is comparable (difference is not statistically significant) with that of the non-fluorinated myristoyl chain (reference compound, BPS1), which is cleaved most efficiently (*K*_cat_/*K*_m_ = 716.5 ± 72.8/s mol). In contrast, the catalytic efficiency of SIRT2 was significantly lower for the fluoropalmitoyl group (*K*_cat_/*K*_m_ = 60.4 ± 7.3/s mol). These observations agree with previous reports, demonstrating a similar trend in SIRT2 catalytic efficiency for the H3K9 peptide backbone derivatized with hexanoyl, octanoyl, decanoyl, dodecanoyl, and myristoyl groups, but significantly less efficiently the palmitoyl and lipoyl-derivatized H3K9 peptide [23, 24]. Similarly, using TNF-α peptide backbone with varying chain lengths to a fluorophore or quencher moiety the longer chain length (11 carbons) resulted in much improved catalytic efficiency over the shorter (6 or 8 carbons) or very long (13 carbons) chain lengths [25]. However, despite the similarities in trends, the absolute *K_cat_/K_m_* values obtained in the current study for different alkyl chains with Boc-Lys-AMC backbone are significantly lower than those reported previously using a H3K9 or TNF-α peptide [23–25], which can be explained, at least in part, by the higher binding efficiency of a peptide “cap” group, as compared to Boc-Lys-AMC. Similarly, reports of a “cap” group containing either p53 or TNF-α peptide residues on one side and an AMC group on the other demonstrate reduced *K*_cat_/*K*_m_ as compared to compounds with a full peptide cap [23, 25, 26]. Also, the observed differences may be due to the presence of electronegative ω-terminal fluorine. In the current study, the *K*_cat_/*K*_m_ values were determined by measuring the rate of the AMC cleavage product formation [26], whereas in some of the previously reported data, *K*_cat_/*K*_m_ values were determined by measuring the rate of NAD^+^ consumption, which may also explain the differences in absolute *K*_cat_/*K*_m_ values between studies [23].

To examine the selectivity of compounds for SIRT2 among all SIRT enzymes, three of the most efficient substrates, **4**, **5**, and **7** were screened against a panel of recombinant SIRT enzymes (SIRT1–7). The aim was to elucidate the differences in SIRT selectivity that may occur as a result of altering the cap (mono- *vs* di-lysine cap), the leaving group (12- *vs* 14-carbon chain), and last the terminal fluorine atom (12F-carbon *vs* 14-carbon). Our results indicate that compound **7**, with a di-lysine cap, was cleaved more effectively by SIRT3 and SIRT6 than compound **5**, with a mono-lysine cap. Therefore, **5** is more selective to SIRT2 as it shows very little cleavage by other SIRT enzymes. Both **4** (12F-carbon chain) and **5** (14carbon chain) are cleaved effectively by SIRT2 and not by any other SIRT enzymes, indicating that both leaving groups are selective for SIRT2. These findings are consistent with other reports in the literature [22, 24, 27] and demonstrate that leaving group specificity may not entirely define the selectivity of a substrate to a certain SIRT enzyme. Altering the cap group can have a significant impact on the efficiency with which the SIRT enzyme cleaves a substrate.

Also, we assessed the effectiveness of the lead compound **4** as a synthetic substrate for SIRT2 using a potent selective SIRT2 inhibitor AGK2 [28]. A competitive inhibition study [28] was performed using increasing concentrations of AGK2 with **4** at its apparent *K*_m_ concentration (8 μM) for SIRT2. The observed inhibition of SIRT2 by AGK2 at IC_50_ of 28.21 μM using **4** as substrate was comparable to previously reported IC_50_ values of 3– 5 μM obtained with myristoylated or acetylated substrates of SIRT2 [29, 30]. As demonstrated by the comparable catalytic efficiencies of **4** and **BPS1** (p53 (379–382)K382ac-AMC) this data further confirm that **4** is an efficient substrate of SIRT2.

Based on the results of biochemical and *in silico* modeling studies (additional discussion can be found in Supplemental Materials), we have chosen the dodecanoyl chain fluorinated on the ω-position for coupling to an AHA backbone (Fig. S2 ESM) to generate the SIRT2specific ^18^F-labeled radiotracer termed 12-[^18^F]DDAHA. We did not study the 14-fluoromyristoyl-Boc-Lys-AMC and 14-[^18^F]fluoromyristoyl-AHA, because Boc-Lys(12fluorododecanoyl)-AMC exhibits 2/3 efficiency of Boc-Lys(myristoyl)-AMC as selective substrate for SIRT2 and because radiotracers with smaller molecular weight have pharmacokinetic advantages over radiotracers with larger molecular weight. The AHA backbone was chosen to increase the lipophilicity of the radiotracer and permeability across cell membranes, which would not be possible with a larger peptidic backbone. Accordingly, the radiotracer 12-[^18^F]DDAHA has a cLogP value of 5.32 indicating a strong ability to passively cross the cell membrane in and out of the cell. While the change in “cap” group may alter the selectivity to SIRT2, it has a lesser effect on the catalytic efficiency, as this is determined largely by the leaving group structure rather than the cap group. Nevertheless, the synthesis and evaluation of these compounds are currently underway and will be reported separately. For synthesis of 12-[^18^F]DDAHA, we adapted methods for radiolabeling of ω-[^18^F]substituted long-chain fatty acids [19] that are used for PET imaging of cardiac metabolism [31]. Our studies confirmed previous reports that ω-iodinated dodecanoyl precursor provides a 3-fold higher radiosynthetic yield than the ω-brominated precursor [19]. The radiosynthetic yield for 12-[^18^F]DDAHA could be optimized further by using microwave-assisted heating [19].

*In vitro* radiotracer uptake studies in cell lines with varying degrees of SIRT2 expression provided initial evidence for the efficacy of 12-[^18^F]DDAHA as a radiotracer for quantification of SIRT2 expression-activity. The level of 12-[^18^F]DDAHA-derived radioactivity accumulation was significantly increased in MDA-MB-435 melanoma and MCF-7 breast adenocarcinoma and to a much lesser degree, in U87MG glioma, as compared to MiaPaCa pancreatic carcinoma and MCF10A immortalized breast epithelial cells. MCF-7 cells are known to have increased SIRT2 expression [20, 32]. Also, MiaPaCa cells have demonstrated increased levels of SIRT2 expression *via* Western blot analysis [11]. The non-tumorigenic epithelial cells, MCF10A, exhibit significantly lower levels of SIRT2 expression [20] and served as a lower-expressing control for this study. These results indicate levels of 12-[^18^F]DDAHA-derived radioactivity accumulation correspond to levels of SIRT2 expression as reported elsewhere for MCF-7, MDA-MB-435, U87MG, MiaPaCa, and MCF10A cell lines.

Despite the high lipophilicity of the fluoroalkyl leaving group, the mechanism of substrate cleavage by SIRT enzymes allows for a radioactive leaving group to be entrapped in the cell, at least transiently. Specifically, SIRT2 enzymatic cleavage mechanism results in formation of an ester linkage between the alkyl leaving group and *O*-ADPR (Fig. S1 ESM), resulting in ^18^F-fluoroalkyl-*O*-ADPR product which cannot cross a cell membrane because it is quite large and very polar (containing 2 negatively charged phosphate groups) (Fig. S1 ESM). Therefore, SIRT2-mediated cleavage of ^18^F-fluoroalkyl group from 12[^18^F]DDAHA with formation of ^18^F-fluoroalkyl-*O*-ADPR complex represents the rate-limiting step in the transient cellular entrapment of 12-[^18^F]DDAHA-derived radioactivity. The eventual efflux of 12-[^18^F]DDAHA-derived radioactivity from intracerebral 9L glioma also suggests that ^18^Ffluoroalkyl-*O*-ADPR is degradable and that ^18^F-fluoroalkyl moiety eventually diffuses out of the cells. The removal of the acetyl or longer acyl chain moieties from *O*-ADPR group has been hypothesized by others to occur by way of ARH3 cleavage or possible methanolysis to form 1′*O*-methyl′ ADPR [33, 34]. Additionally, a nucleophilic attack from lysine side chains of other proteins in close proximity may also de-acetylate the *O*-AADPR. Further studies are needed to fully characterize the half-life of *O*-12-[^18^F]DD-ADPR and residence time for the 12-[^18^F]dodecanoyl moiety within the cell. Although this entrapment may be transient, it provides a sufficient retention time within the cells with high SIRT2 expression-activity for PET imaging.

Furthermore, our initial *in vivo* PET imaging studies using 12-[^18^F]DDAHA conducted in a 9L glioma-bearing rats were discouraging, due to rapid systemic defluorination of this radiotracer upon intravenous administration. This was evidenced by significant accumulation of F-18 radioactivity in the skull and other bones, which confounded the interpretation of images of radiotracer accumulation within the tumor and other regions of the brain. Therefore, additional PET/CT MR images in panel Fig. 4d are shown in NIH + white (max) look-up table, and the resulting PET/ MRI fusion images revealed 12-[^18^F]DDAHA-derived radioactivity accumulation in 9L gliomas and normal brain regions, such as the hippocampus. This proof of concept imaging study demonstrated adequate BBB permeability, as evidenced by the rapid equilibration of 12-[^18^F]DDAHA radioactivity between blood and normal brain tissue (Fig. 4). Progressive accumulation of 12-[^18^F]DDAHA-derived radioactivity in normal brain structures with high levels of SIRT2 expression and especially in 9L tumor lesions (Fig. 4) during the first 15-min post intravenous administration supports the proposed mechanism of SIRT2-mediated accumulation and transient retention in tissues. Static images of 12-[^18^F]DDAHA accumulation/retention (SUV) obtained between 15 and 20 min post i.v. injection with highest tumor-to-normal brain and tumor-to-muscle SUV ratios may provide more specific images of SIRT2 expression-activity in target tissues, if defluorination would be minimized in the next generation of radiotracers. Because of the observed excessive defluorination of 12-[^18^F]DDAHA upon intravenous administration, a more complete characterization of this radiotracer *in vivo* (*i.e*., blocking studies) has not been performed.

To validate the results of PET/CT/MR imaging with 12[^18^F]DDAHA and to assess the cell specificity and heterogeneity of SIRT2 expression-activity in 9L gliomas and in different brain structures, we used immunofluorescent microscopy (IFM) in 9L glioma-bearing rat brain tissue sections. Fluorescence microscopy of tissue sections of intracerebral 9L gliomas growing in the rat brains stained with anti-SIRT2 fluorescent antibodies revealed heterogeneously distributed patchy-appearing circular regions of SIRT2 expression in core regions in peripheral infiltrating zones of 9L gliomas. Such pattern of expression suggested that SIRT2 is upregulated in well-perfused regions of 9L gliomas, whereas the expression of SIRT2 in hypoxic-appearing regions is significantly reduced, which was confirmed immunohistochemically by presence of HIF-1α-expression in the same regions in adjacent tissue sections. These results are consistent with previous reports of regulatory loops of HIF-1a-SIRT2, involving HIF-1α-mediated transcriptional repression of SIRT2 expression [35], as well as SIRT2-mediated deacetylation of Lys709, which increases prolyl-hydroxylase binding and ubiquitin-mediated degradation of HIF-1α [36].

In the normal brain structures, the highest level of SIRT2 expression was observed in hippocampal CA2 and CA3 neurons, followed by pyramidal neurons in cerebral cortex, and cerebellar Purkinje cells. The subcellular localization of SIRT2 was predominantly perinuclear with lower levels of diffuse distribution in the rest of the neuronal cytoplasm and axons. These observations of neuronal-specific subcellular localization of SIRT2 in the brain are in agreement with previous reports [21] which demonstrated that SIRT2 expression in the murine brain is higher in hippocampal CA2–3 neurons, cortical neurons, and in cerebellar Purkinje cells and molecular layer neurons, but not in the granular layer neurons or other cells of oligedendroglial lineage. Thus, *in situ* microscopy results can serve as the basis for interpretation of *in vivo* PET/CT/MRI images and confirm that the increased levels of radiotracer accumulation in intracerebral 9L gliomas and in certain normal brain structures are mediated by increased SIRT2 expression-activity levels, as compared to other brain structures expressing lower levels of SIRT2.

Also, this study provides grounds for development of the next generation of SIRT2-specific radiotracers. Several previous studies have reported defluorination of ω[^18^F]substituted fatty acids in rodents following intravenous injection [31] but significantly lower levels of defluorination occurred in higher order mammals (*i.e*. pigs and dogs) and in humans, due to a decrease in level of expression of defluorinating enzymes [31]. An alternative approach to decrease defluorination in a second-generation tracer would be to radiolabel at the ω−3 position of the acyl chain. Additionally, carbon-11 labeling of the dodecanoyl chain could be accomplished similar to ^11^C-palmitate [37, 38]. Another approach to decrease the magnitude of systemic defluorination could involve pre-treatment with miconazole (CYP450 2E1 inhibitor) which was shown effective for ^18^F-FCWAY [39], which, however, is not practical for clinical translation because of potential for high patient to patient variability. Further radiotracer development is underway and will be reported elsewhere.

SIRT2 is an emerging target for therapy of various neurologic diseases, including neurodegeneration [10], as well as cardiac [40] and metabolic diseases [40, 41]. Many SIRT2-targeted inhibitors are currently in development and entering the early-stage clinical trials, further driving the need for continuation of this work towards development and clinical translation of second-generation SIRT2-selective radiotracer.

## Conclusions

In summary, guided by the results of biochemical and *in silico* modeling studies of structure-activity relationships between SIRT2 and a peptide-mimetic backbone containing a lysine derivatized with fluoroalkyl chains of different length, we have developed and tested *in vitro* and *in vivo* a novel, first-generation, SIRT2-selective substrate-type radiotracer, 12-[^18^F]DDAHA. Current proof of principal studies, demonstrated suitability for radiofluorinated DDAHA assessment of SIRT2 expression-activity using *in vitro* radiotracer uptake assays. Molecular imaging with PET using next-generation SIRT2-specific radiofluorinated DDAHA-based radiotracer, which is resistant to defluorination *in vivo*, should provide valuable information about the location and magnitude of SIRT2 expression and activity in the brain and in tumors. PET imaging may help to determine the mechanistic, therapeutic, and prognostic roles of SIRT2 in different diseases and enable monitoring of SIRT2 targeted therapies.

## Supplementary Material

Supplement

## Figures and Tables

**Table 1.** Kinetic parameters for SIRT2 and various chain length compounds

Compound	*K*_m_ (μM)	SD	*V*_max_ (μM/s)	SD	*K*_cat_ (/sec)	SD	*K*_cat_/*K*_m_ (/s mol)	SD

Boc-Lys(3-F-propanoyl)-AMC (**1**)	55.53	11.4	0.15	0.016	1.41 × 10^−3^	5.0 × 10^−4^	26.3	5.1
Boc-Lys(6-F-hexanoyl)-AMC (**2**)	44.89	4.56	0.28	0.013	2.58 × 10^−3^	4.1 × 10^−4^	57.4	5.2
Boc-Lys(10-F-decanoyl)-AMC (**3**)	18.03	4.07	0.52	0.041	4.86 × 10^−3^	1.3 × 10^−4^	269.5	52
Boc-Lys(12-F-dodecanoyl)-AMC (**4**)	8.07	0.79	0.54	0.014	4.97 × 10^−3^	4.2 × 10^−4^	615.4	50
Boc-Lys(myristoyl)-AMC (**5**)	2.36	0.22	0.26	0.010	2.38 × 10^−3^	3.0 × 10^−4^	715.5	73
Boc-Lys(palmitoyl)-AMC (**6**)	51.15	6.91	0.33	0.022	3.09 × 10^−3^	6.8 × 10^−4^	60.4	7.3
Cbz-Lys-Lys(myr)-AMC (**7**)	10.36	1.30	0.50	0.022	5.51 × 10^−3^	6.9 × 10^−4^	532.3	56
BPS1	31.73	5.57	0.95	0.022	8.78 × 10^−3^	7.0 × 10^−4^	275.0	43
