# Supplementary material for: A Novel Substrate Radiotracer for Molecular Imaging of SIRT2 Expression and Activity with Positron Emission Tomography
Source: Mol Imaging Biol. Author manuscript; Available in PMC 2019 Oct 28. (PMC6816246; doi:10.1007/s11307-017-1149-8)
Supplement: Supplement [file NIHMS1054198-supplement-Supplement.pdf]

Electronic Supplementary Material

A Novel Substrate Radiotracer for Molecular Imaging of SIRT2 Expression and Activity with Positron Emission Tomography.

Robin E. Bonomi[1], Maxwell Laws[1], Vadim Popov[1], Swatabdi Kamal[1], Shreya Potukutchi[1], Aleksandr Shavrin[1], Xin Lu[2], Nashaat Turkman[1], Ren Shuan Liu[3], Thomas Mangner[2], Juri G. Gelovani[1]*

Journal: Molecular Imaging and Biology

MATERIALS AND METHODS

All solvents were purchased from Sigma-Aldrich Chemicals (Milwaukee, WI) and used without further purification. Boc-Lys(Ac)-AMC and Cbz-Lys-OH were purchased from Bachem (Bubendorf, Switzerland) and used without further purification. Preparation of 6-amino-1-hexanoicanilide[1] was synthesized using a previously published procedure[2]. Thin layer chromatography (TLC) was performed on pre-coated Dynamic Absorbance F-254 silica gel, aluminum backed plates (Norcross, Georgia); flash chromatography was performed using silica gel pore size 60 Å, 230-400 mesh particle size (Sigma-Aldrich, Milwaukee, WI).

NMR spectra including ^{1}H, ^{13}C and ^{19}F were performed on Mercury 400 MHz, Varian 500 MHz or Agilent 600 MHz spectrometers. High-resolution mass spectra (HRMS) were obtained using Waters LCT Premier/XE spectrometer (Milford, MA) with an electrospray ionization (ESI) technique. The analytical high performance liquid chromatography (HPLC) system included: Ascentis RP-Amide 4.4x150 mm column (Supelco, Bellefonte, PA) connected to the 1100 series pump and UV detector (Agilent Technologies, Stuttgart, Germany), operated at 254 nm, and a radioactivity detector FC3200 (Eckert and Ziegler Radiopharma, Inc, Berlin, Germany). The semi-preparative HPLC system included: the Alltima 250x10mm C18 column (Fisher Scientific, Waltham, MA) connected to the P4.1S pump, Azura 2.1S UV detector (Knauer, Berlin, Germany), operated at 254 nm, and a radioactivity detector FC-3500 (Eckert and Ziegler Radiopharma, Inc, Berlin, Germany).

Synthesis of ω-fluorinated acyl chains. 3-fluoropropionic acid was purchased from Santa Cruz Biotechnology (Dallas, TX) and used without further purification. 10-hydroxydecanoic acid, 12-

hydroxydodecanoic acid, and 16-hexadecanoichydroxyhexadecanoic acid were purchased from Sigma Aldrich (Milwaukee, WI) and used for synthesis of ω-fluorinated carboxylic acid chains. The n-hydroxycarboxylic acids were treated with molar excess of diethylaminosulfur trifluoride (DAST) at 0°C for 5-10 minutes. Immediately following, the reaction mixture was transferred dropwise to 5 ml of hexane resting on a small plug of silica gel primed with hexane to selectively hydrolyze the acetyl fluoride formed during the DAST reaction. The compounds were eluted from the silica using 50:50 ethyl acetate/hexane and they were used without further purification for formation of acetyl chloride and coupling to the lysine derivative. Further details of synthesis of compounds **1-14** can be found in supplemental materials (Supplemental Data).

SIRT Enzyme Assay. Recombinant SIRT1-7 enzymes and BPS1 reference substrate for SIRT2, an oligopeptide corresponding to 379-382 of p53 (Arg-His-Lys-Lys(Ac)-AMC)[3] were purchased from BPS Bioscience (San Diego, CA). 10 mM solutions of all newly synthesized compounds were prepared in pure DMSO and then diluted to 100 μM aliquots in DMSO for further use. Enzyme assay reactions were performed in black, low binding 96-well microtiter plates (Nagle Nunc International, Rochester, NY). End point assays were performed by incubation of the appropriate substrate, NAD, a SIRT enzyme, and inhibitor (if applicable) in enzyme assay buffer (50 μL final volume). Control wells without enzyme were included in each plate. After 40 min at 37 °C, a developer mixture (50 μL) containing nicotinamide (2 mM) and trypsin (0.4 mg mL^{-1}) was added, and the mixtures incubated for 30 min at 22°C. The intensity of cleaved 7-amino-4-methylcoumarin (AMC) fluorescence was measured using Synergy H1 microplate reader (Biotek, Winooski, VT) at 360 nm excitation and 450 nm emission wave lengths. Experiments for determination of enzyme kinetic parameters were performed by incubation of test compounds or BPS1 reference substrate at different concentrations with NAD^+ and individual SIRT enzymes in

an assay buffer (100 μL final volume) at 37°C for different periods of time (5, 10, 20, 30, and 60 min), followed by *in situ* fluorophore cleavage by addition of a developer mixture (50 μL) containing nicotinamide (2 mM) and trypsin (0.4 mg mL^{-1}). Fluorescence was measured as relative fluorescence units (RFU) at 22°C to determine the initial linear rate V_0 (RFU min^{-1}) for each concentration. The data were analyzed using GraphPad Prism v6.02 (GraphPad, La Jolla, CA) to afford k_m (μM) and V_{max} (RFU min^{-1}) values. The k_{cat} values were calculated based on the RFU vs AMC concentration standard curve and the enzyme purities and concentrations provided by the manufacturer.

Computational Docking Studies. Schrodinger Suite (New York, NY) with the Glide[4] docking program was used for simulating ligand-protein interactions *in silico*. The ligands were prepared using LigPrep[5] in Maestro v10.3 and the lowest 5 energy states were used for covalent docking interactions. Epik was used to generate the tautomeric states and choosing the lowest Epic[6] ionization state. The protein, SIRT2 (PDB: 4RMG) was prepared with the Protein Preparation Wizard[5]. A Cartesian coordinate receptor grid was generated on SIRT2 by positioning the prereaction form of the ligand in the binding site close to the NAD^+. The affinity score is given as a Glide score based on the binding mode of the ligand with the protein where the optimal interactions are represented by a low Glide score[7]. The distance between the NAD^+ and the carbonyl-carbon of the leaving group cleavage site was calculated manually using the Measure Distance functionality.

Cell cultures and in vitro Radiotracer Uptake Studies. The U87MG, MCF-7, MDA-MB-435, and MiaPaca cell lines were obtained from the American Type Tissue Culture Collection (ATCC); the MCF10A cell line was obtained from the cell culture repository of the Karmanos Cancer Institute (Detroit, MI). Tumor cells were propagated in DMEM supplemented with 10% fetal calf

serum (FCS), penicillin and streptomycin, at 37°C in a humidified atmosphere with 5% CO_2. In vitro radiotracer uptake studies were performed as previously described[8]. Briefly, prior to in vitro radiotracer uptake experiment, the cells were inspected for confluency and viability. Each of the four cell plates for a given cell line had the media removed by pipette aspiration, and fresh media (15mL) containing the radiotracer (1-2uCi/mL) was added to cells in the culture plates. The tumor cell monolayers were incubated with the radioactivity-containing medium for 30 minutes. The cells were then harvested by gentle unidirectional scraping and the suspensions of cells in the media were transferred into 15mL conical centrifuge tubes and pelleted by centrifugation (3000 rpm for 2 min). 50uL samples of supernatant and cell pellets flash-frozen on dry ice were placed in pre-weighed scintillation vials, weighed, and the radioactivity was measured using Pakard 5500 gamma counter (Perkin Elmer, CA). The background-corrected and decay-corrected radioactivity concentrations in individual cell pellets (cpm/g) was divided by corresponding cell culture media samples (cpm/g) to determine the fold increase of radiotracer accumulation in cells versus the cell culture media. All experiments were conducted in triplicate.

Intracerebral glioma model in rats. All studies in rats were performed under a protocol approved by the Institutional Animal Care and Use Committee of Wayne State University. The 9L cells were obtained from ATCC and propagated in MEM supplemented with non-essential amino acids and 10% FCS in at 37°C in humidified atmosphere with 5% CO_2. For preparation of cellular suspension for intracerebral injection, the 9L cells were dislodged from cell culture flasks using Hank's Balanced Salt solution (HBSS, 5 mL) for 1-2 minutes, centrifuged to obtain the cellular pellet, and re-suspended in cell culture medium without FCS at $1x10^4$ cells/µL concentration. Sprague-Dawley rats (200-250 g, N=3) were anesthetized by inhalation of isoflurane in oxygen (3-4% isoflurane for induction; 1.5-2.5% isoflurane for maintenance). The body temperature was

maintained using electronically-controlled heating pad (M2M Imaging, Cleveland, OH) set at 37°C. The rats were placed into a stereotaxic apparatus (Kopf Tujunga, CA). A midline scalp incision was made to reveal bregma and a burr hole 2 mm in diameter was drilled in the skull till *dura mater* and the bone hemostasis was achieved by application of bone wax. Then, a short-beveled 30-ga needle connected to a 100 µL syringe (Hamilton, CA) loaded with the cell suspension was inserted in to the brain using the following stereotactic coordinates relative to *bregma*; AP: -1.5, LAT: -4, DV: -6. A 5 µL tumor cell suspension was then injected slowly over 10 minutes, then the needle was slowly withdrawn 2 mm and the remaining 5 µL was injected slowly over 10 minutes for a total of $1x10^5$ cells in 10 µL.

MR Imaging. The rats were anesthetized and body temperature was maintained at 37°C, as described above. The animals' head was fixed in position using a bite bar and ear bars with a receive-only surface coil 2-element phased array placed dorsally on top of the head. MR images were acquired using a 7T ClinScan system (Bruker, UK) controlled by the Syngo software (Siemens, Knoxville, TN). A localizing T1-weighted scan was performed and adjustments to head position were made as needed. T_2 images were obtained with TR 3530 ms, TE 38 ms, and FOV 3.2 cm x 3.2 cm x 2.4 cm, resulting in spatial resolution of 125 µm x 125 µm x 1 mm. Images were processed using ImageJ software (NIH, Bethesda, MD).

PET/CT Imaging. The rats were anesthetized and maintained under anesthesia throughout the PET/CT imaging studies, as described above for MRI and surgical procedures. Anesthetized rats were placed in stereotactic head holder made of polycarbonate plastic (Kopf-Tujunga, Germany) and attached to the bed the microPET R4 scanner (Siemens, Knoxville, TN) in the supine position with the long axis of the animal parallel to the long axis of the scanner and the brain positioned in the center of the field of view (FOV). [^{18}F]-12FDDAHA (300-500 µCi/animal) was administered

in saline via the tail-vein in a total volume ≤1ml, as a slow bolus injection over the period of 1 min. Dynamic PET images were obtained over 60 minutes. After PET imaging, the positioning bed with the affixed anesthetized animal was transferred to the Inveon SPECT/CT scanner (Siemens, Knoxville, TN) and CT images and 4 overlapping frames (2 min each) were acquired covering the whole body using X-ray tube settings of 80 kV and 500 uA with exposure time of 300–350 milliseconds of each of the 360 rotational steps.

Histology and Immunohistochemistry. After the completion of in vivo imaging studies, the animals were euthanized under anesthesia by decapitation using a small animal guillotine. The brain was extracted, frozen in isopentane bath on dry ice, embedded in OCT, sectioned at 20 µm using OTF5000 cryomicrotome (Hacker-Bright, SC), mounted on glass slides and post-fixed in 4% parafolmaldehyde overnight. To visualize the SIRT2 expression, tissue sections were washed in TBS, heat-treated at 90°C in citrate buffer for 45 min, blocked with 5% normal horse serum for 30 min, washed in TBS again and incubated overnight at 4°C with a 1:100 diluted mouse monoclonal antibody against SIRT2 conjugated with AlexaFluor 488 (clone A-5; catalogue number sc-28298, Santa Cruz Biotechnology, CA). To visualize HIF-1α in adjacent tissue sections, first, the tissue sections were heat-treated at 90°C in citrate buffer for 45 min; then, endogenous peroxidase activity was quenched by incubation in 3% H_2O_2 in PBS; then tissue sections were then washed in PBS and blocked by 10% normal horse serum and incubated with 1:100 diluted mouse monoclonal antibody against HIF-1α (NB100-123, Novus Biologicals, CO), followed by incubation with 1:200 diluted secondary horse-anti-mouse biotinylated antibodies and avidin-peroxidase (Vectastain Elite Kit, Vector Laboratories, CA) and finally with 3,3-diaminobenzidine chromophore in water with 2% H_2O_2. Adjacent tissue sections were counter-stained with H&E.

Chemistry Methods:

tert-Butyl (7-(3-fluoropropanamido)-1-((4-methyl-2-oxochroman-7-yl)amino)-1-oxoheptan-2-yl)carbamate (1)

3-Fluoropropionic acid (61.7 mg, 0.66 mmol) was dissolved in acetonitrile with 1 equivalent of *N*,*N'*-dicyclohexylcarbodiimide (DCC) (155 mg) and a catalyst amount of 4-dimethylaminopyridine (DMAP) (few crystals). The mixture was stirred at 22°C under argon for 10 minutes. Subsequently, Boc-Lys-AMC (188 mg, 0.46 mmol) was dissolved in 7 mL of dichloromethane (DCM) with 1 equivalent of triethylamine (TEA) and added dropwise to the reaction mixture. The reaction was stirred continuously at 22°C, under argon, for 12 hours. Following completion of the reaction, the solvent was evaporated and the compound was purified by column chromatography using a gradient of 0.5-2% methanol/dichloromethane for elution. The product was obtained in 85% yield as an off-white powder. ^{1}H NMR (400 MHz, $CDCl_3$) δ 9.42 (s, 1H), 7.68 (s, 1H), 7.47 (s, 1H), 7.38 (s, 1H), 7.26 (s, 1H), 6.20 (s, 1H), 6.14 (s, 1H), 5.54 (s, 1H), 5.29 (s, 1H), 4.79 (t, J = 5.6 Hz, 1H), 4.67 (t, J = 5.6 Hz, 1H), 4.30 (s, 1H), 3.51 – 3.37 (m, 1H), 3.35 – 3.16 (m, 1H), 2.60 (dd, J = 15.3, 9.7 Hz, 1H), 2.54 (t, J = 5.6 Hz, 1H), 2.38 (s, 2H), 1.45 (s, H), 1.94 – 1.88 (m, 2H), 1.82 – 1.62 (m, 2H), 1.57 (dd, J = 16.4, 11.8 Hz, 2H), 1.30 (dt, J = 17.4, 13.0 Hz, 2H), 1.20 – 0.99 (m, 2H). ^{19}F NMR (376 MHz, $CDCl_3$) δ -217.96 (tt, J = 47.0, 27.5 Hz). HRMS (+ESI-TOF) m/z for $C_{25}H_{35}N_3O_6$ $[M+Na]^+$ calculated: 493.5802; found: 493.2097

tert-Butyl (7-(6-fluorohexanamido)-1-((4-methyl-2-oxochroman-7-yl)amino)-1-oxoheptan-2-yl)carbamate (2)

6-Bromoethylhexanoate (1 g) was dissolved in 10 mL of TBAF and stirred at 80°C for 3 hours. The resulting mixture was evaporated and purified by column chromatography using 10% ethyl acetate in hexane as the eluent. The ethyl protecting group was hydrolyzed by 100ul of 15wt% sodium methoxide in methanol. The following mixture was evaporated and 2 mL of thionyl

chloride ($SOCl_2$) was added to the residue. The resulting mixture was stirred at 60°C, under reflux, for 3 hours. Following the reaction, all $SOCl_2$ was evaporated and the product was washed with toluene before being evaporated once more. Boc-Lys-AMC (Bachem (92 mg, 0.24 mmol) was dissolved in 10 mL of dichloromethane with 1 equivalent of TEA. The mixture was added dropwise to the flask containing the acetyl chloride residue, and the reaction was stirred continuously at 22°C, under argon, for 12 hours. Following completion of the reaction, the solvent was evaporated and the compound was purified by column chromatography using a gradient of 3-5% methanol/dichloromethane for elution. The product was obtained in 20% yield as an off white powder. ^{1}H NMR (600 MHz, $CDCl_3$) δ 11.20 (s, 1H), 9.40 (s, 1H), 7.68 (s, 1H), 7.45 (s, 2H), 7.42 (s, 2H), 7.25 (d, 2H), 6.13 (s, 1H), 5.95 (s, 1H), 5.50 (s, 1H), 4.45 (t, J = 5.9 Hz, 1H), 4.37 (t, J = 5.7 Hz, 1H), 4.28, 3.33 – 3.26 (m, 1H), 3.23 (dd, J = 13.3, 6.7 Hz, 1H), 2.99 (d, J = 4.3 Hz, 1H), 2.55 (s, 2H), 2.37 (s, 2H), 2.18 (t, J = 7.4 Hz, 2H), 1.77 – 1.62 (m, 7H), 1.57 (dd, J = 13.8, 6.9 Hz, 3H), 1.50 – 1.40 (m, 14H), 0.95 (t, J = 7.1 Hz, 2H). ^{13}C NMR (151 MHz, $CDCl_3$) δ 173.2, 171.4, 161.1, 154.5, 152.3, 141.5, 125.0, 123.6, 115.8, 115.6, 113.2, 107.1, 83.9 (d, J = 164.4 Hz), 80.4, 77.0 76.8, 60.0, 55.0, 52.2, 48.6, 38.3, 36.5, 31.2, 30.1 (d, J = 19.5 Hz), 28.8, 28.3, 25.3, 25.1, 25.0, 24.9, 22.5, 20.1, 18.5, 13.5. ^{19}F NMR (376 MHz, $CDCl_3$) δ -175.21 – -184.59 (m). HRMS (+ESI-TOF) m/z for $C_{28}H_{41}N_3O_6$ $[M+Na]^+$ calculated: 535.66; found: 535.2642

***tert*-Butyl (7-(10-fluorodecanamido)-1-((4-methyl-2-oxochroman-7-yl)amino)-1-oxoheptan-2-yl)carbamate (3)**

10-Fluorodecanoic acid (45 mg, 0.24 mmol) was dissolved in excess $SOCl_2$ (1 mL, 1.3 mmol) and stirred at 60°C, under reflux, for 3 hours. Following the reaction, all $SOCl_2$ was evaporated and the product was washed with toluene before being evaporated once more. Boc-Lys-AMC (96 mg, 0.24 mmol) was dissolved in 7 mL of dichloromethane with 1 equivalent of TEA. The mixture

was added dropwise to the flask containing the acetyl chloride residue, and the reaction was stirred continuously at 22°C, under argon, for 12 hours. Following completion of the reaction, the solvent was evaporated and the compound purified by column chromatography using a gradient of 3-5% methanol/dichloromethane for elution. The product was obtained in 55% yield as an off white powder. ^{1}H NMR (499 MHz, $CDCl_3$) δ: 9.55 (s, 1H), 7.70 (s, 1H), 7.45 (s, 2H), 7.33 (s, 1H), 6.13 (s, 1H), 5.94 (s, 1H), 5.59 (s, 1H), 4.47 (d, J = 12.1 Hz, 1H), 4.39 – 4.34 (m, 1H), 4.02 (s, 1H), 2.38 (s, 2H), 1.95 (s, 2H), 2.28 (t, J = 7.5 Hz, 1H), 2.16 (t, J = 7.6 Hz, 2H), 1.79 – 1.52 (m, 9H), 1.46 (s, 9H), 1.26 (s, 10H). ^{13}C NMR (126 MHz, $CDCl_3$) δ 173.6, 171.6, 161.2, 156.5, 154.0, 152.5, 141.6, 125.0, 115.7, 115.6, 113.1, 107.1, 84.2 (d, J = 163.9 Hz), 80.4, 64.3, 55.1, 38.5, 36.7, 34.4, 31.6, 30.4, 30.3, 29.4, 29.31, 29.26, 29.2, 29.1, 29.0, 28.6, 28.4, 25.8, 25.7, 25.13, 25.08, 25.0, 22.7, 18.5. ^{19}F NMR (376 MHz, $CDCl_3$) δ -217.73 – -218.25 (m). HRMS (+ESI-TOF) m/z for $C_{32}H_{49}N_3O_6$ $[M+Na]^+$ calculated: 614.3519, found M+Na: 614.3581

tert-Butyl (7-(12-fluorododecanamido)-1-((4-methyl-2-oxochroman-7-yl)amino)-1-oxoheptan-2-yl)carbamate (4)

12-Fluorododecanoic acid (21 mg, 0.027 mmol) was dissolved in excess $SOCl_2$ (1 mL, 1.3 mmol) and stirred at 60°C, under reflux, for 3 hours. Following the reaction, all $SOCl_2$ was evaporated and the product was washed with toluene before being evaporated once more. Boc-Lys-AMC (36 mg, 0.09mmol) was dissolved in 7 mL of dichloromethane with 1 equivalent of triethylamine (TEA). The mixture was added dropwise to the flask containing the acetyl chloride residue, and the reaction was stirred continuously at 22°C, under argon, for 12 hours. Following completion of the reaction, the solvent was evaporated and the compound was purified by column chromatography using a gradient of 3-8% methanol/dichloromethane for elution. The product was obtained in 20% yield as an off white powder. ^{1}H NMR (500 MHz, $CDCl_3$) δ: 9.55 (s, 1H), 7.70

(s, 1H), 7.43 (d, J = 8.4 Hz, 1H), 7.32 (d, J = 7.5 Hz, 1H), 6.12 (s, 1H), 5.95 (s, 1H), 5.62 (d, J = 6.5 Hz, 1H), 4.41 (dt, J = 47.4, 6.2 Hz, 2H), 3.34 – 3.16 (m, 2H), 2.36 (s, 3H), 2.15 (t, J = 7.7 Hz, 2H), 1.99 – 1.88 (m, 2H), 1.81 – 1.71 (m, 2H), 1.72 – 1.51 (m, 8H), 1.44 (s, 9H), 1.39 – 1.32 (m, 4H), 1.23 (d, J = 5.6 Hz, 14H). ^{13}C NMR (126 MHz, $CDCl_3$) δ: 173.8, 171.8, 161.3, 156.6, 154.1, 152.6, 141.7, 125.1, 115.8, 115.7, 113.2, 107.2, 84.3 (d, J = 163.8 Hz). 80.5, 55.3, 38.7, 36.9, 31.7, 30.6, 30.4, 29.6, 29.4, 29.3, 29.1, 28.5, 25.9, 25.3, 25.2, 25.0, 22.8, 18.6. ^{19}F NMR (376 MHz, $CDCL_3$) δ -217.99 (dq, *J* = 47.4, 25.0 Hz). HRMS (+ESI-TOF) m/z for $C_{34}H_{53}N_3O_6$ $[M+Na]^+$ calculated: 626.3415 found: 626.3395

tert-Butyl (7-(14-decanamido)-1-((4-methyl-2-oxochroman-7-yl)amino)-1-oxoheptan-2-yl)carbamate (5)

Boc-Lys-AMC (100 mg, 0.25 mmol) was dissolved in 5 mL of dimethylformamide (DMF) with excess of *N*,*N*-diisopropylethylamine (DIPEA) (0.5 mL). Myristoyl chloride (92 mg, 0.37 mmol Sigma-Aldrich, Milwaukee, WI) was added dropwise to reaction at 0°C and subsequently stirred at 22°C under argon for 12 hours. The resulting mixture was evaporated and the residue was dissolved into ethyl acetate (EtOAc) and was washed 2 times with 2N aqueous hydrochloric acid solution. The organic layer was separated and dried for 2 hours on sodium sulfate, evaporated, and purified by using flash chromatography in an Alltech 900 mg silica cartridge (Fisher Scientific, Waltham, MA) with EtOAc as an eluent. The compound was isolated as a white powder in 64% yield. ^{1}H NMR (600 MHz, $CDCl_3$) δ 9.62 (s, 1H), 7.71 (s, 1H), 7.42 (d, *J* = 8.3 Hz, 1H), 7.29 (d, *J* = 7.7 Hz, 1H), 6.14 (s, 1H), 6.10 (s, 1H), 5.67 (d, *J* = 7.0 Hz, 1H), 4.34 (d, *J* = 4.0 Hz, 1H), 3.33 – 3.15 (m, 2H), 2.35 (s, 3H), 2.16 (t, *J* = 7.6 Hz, 2H), 1.95 – 1.87 (m, 1H), 1.79 – 1.69 (m, 1H), 1.61 – 1.53 (m, 4H), 1.44 (s, 9H), 1.31 – 1.15 (m, 26H), 0.85 (t, *J* = 7.0 Hz, 3H). ^{13}C NMR (151 MHz, $CDCl_3$) δ 174.0, 171.8, 161.3, 156.6, 154.0, 152.7, 141.7, 125.1, 115.8, 115.7, 113.2, 107.2,

80.5, 55.3, 38.8, 36.8, 32.0, 31.8, 29.8, 29.8, 29.7, 29.6, 29.5, 29.5, 29.1, 28.5, 26.0, 22.9, 22.8, 18.6, 14.2. HRMS (+ESI-TOF) m/z for $C_{36}H_{57}N_3O_6$ $[M-CH_3+Na]^+$ calculated: 636.3890 found: 636.3795.

tert-Butyl (7-(16-fluorohexadecanamido)-1-((4-methyl-2-oxochroman-7-yl)amino)-1-oxoheptan-2-yl)carbamate (6)

16-Fluorohexadecanoic acid (100 mg, 0.38 mmol) was dissolved in excess $SOCl_2$ (1 mL, 1.3 mmol) and stirred at 60°C, under reflux, for 3 hours. Following the reaction, all $SOCl_2$ was evaporated and the product was washed with toluene before being evaporated once more. Boc-Lys-AMC (100 mg, 0.25 mmol) was dissolved in 10 mL of dichloromethane with 1 equivalent of triethylamine (TEA). The mixture was added dropwise to the flask containing the acetyl chloride residue, and the reaction was stirred continuously at 22°C, under argon, for 12 hours. Following completion of the reaction, the solvent was evaporated and the compound was purified by column chromatography using a gradient of 3-8% methanol/dichloromethane for elution and isolated as a white powder in 20% yield. ^1H NMR (500 MHz, $CDCl_3$) δ 9.27 (s, 1H), 7.69 (s, 1H), 7.50 (s, 2H), 6.19 (s, 1H), 5.76 – 5.67 (m, 1H), 5.40 (d, J = 7.7 Hz, 1H), 5.31 (s, 1H), 4.49 (t, J = 6.2 Hz, 1H), 4.39 (t, J = 6.2 Hz, 1H), 4.06 (t, J = 6.7 Hz, 3H), 3.67 (d, J = 4.8 Hz, 3H), 3.65 (d, J = 4.7 Hz, 1H), 2.41 (s, 2H), 2.30 (dd, J = 15.0, 7.5 Hz, 5H), 2.21 – 2.17 (m, 1H), 1.66 – 1.60 (m, 9H), 1.47 (s, 9H), 1.28 (d, J = 16.1 Hz, 28H). ^{19}F NMR (376 MHz, $CDCl_3$) δ -217.83 – -218.35 (m). HRMS (+ESI-TOF) m/z for $C_{38}H_{61}N_3O_6$ $[M+Na]^+$ calculated: 698.4500, found: 698.4520

Benzyl (6-amino-1-((1-((4-methyl-2-oxochroman-7-yl)amino)-1-oxo-6-propionamidohexan-2-yl)amino)-1-oxohexan-2-yl)carbamate (7)

Boc-Lys-AMC (232 mg, 0.45mmol) was dissolved in trifluoroacetic acid (TFA) to remove the boc protecting group. The mixture was stirred for 30 minutes at 22°C and the TFA (2.5 mL) was evaporated. The resulting residue was dissolved in dichloromethane (DCM) and 1eq of TEA was added to neutralize the product. The mixture was evaporated and the product was washed with water and dried to remove salts. The intermediate product was obtained as 138 mg of white powder. This product was dissolved in DCM and 0.8 equivalents (85 mg) of Cbz-Lys-OH was added along with 1.5 eq. 1-Ethyl-3-(3-dimethylaminopropyl)carbodiimide (EDC) (67 mg) and Hydroxybenzotriazole (HOBT) (50 mg) and 3 eq. of DIPEA (0.12 mL). The mixture was stirred for 12 hours at 22°C under argon. The mixture was evaporated and dissolved in ethyl acetate and washed with saturated sodium carbonate solution. The organic layer dried over magnesium sulfate and evaporated. The residue was purified by column chromatography and the product was eluted with a gradient of 3-5% MeOH/DCM. The product was obtained as an off white powder in 12% yield (40 mg). ^{1}H NMR (600 MHz, DMSO-d_6) δ 10.41 (s, 1H), 8.08 (d, J = 7.4 Hz, 1H), 7.75 (s, 1H), 7.68 (dd, J = 10.8, 7.3 Hz, 2H), 7.46 (d, J = 8.5 Hz, 1H), 7.37 (d, J = 7.7 Hz, 1H), 7.32(s, 3H), 7.28 (dd, J = 7.3, 2.8 Hz, 1H), 6.72 (t, J = 5.5 Hz, 1H), 6.24 (s, 1H), 5.00 (s, 2H), 4.34 (dd, J = 13.7, 7.7 Hz, 1H), 3.98 (dd, J = 12.8, 8.3 Hz, 1H), 3.02 – 2.95 (m, 2H), 2.84 (dt, J = 12.7, 6.5 Hz, 2H), 2.37 (s, 3H), 1.96 (t, J = 7.4 Hz, 2H), 1.75 – 1.65 (m, 1H), 1.65 – 1.53 (m, 2H), 1.48 (d, J = 9.4 Hz, 1H), 1.42 – 1.34 (m, 3H), 1.33 (12H), 1.18 (s, 22H), 0.81 (t, J = 7.0 Hz, 3H). ^{13}C NMR (126 MHz, $CDCl_3$) δ: 174.2, 173.3, 170.9, 161.4, 157.0, 156.7, 154.1, 152.6, 141.7, 136.2, 128.6, 128.3, 128.1, 125.0, 116.0, 113.3, 107.4, 79.4, 67.2, 55.7, 54.1, 50.8, 38.5, 36.9, 32.0, 30.9, 29.8, 29.7, 29.6, 29.5, 29.4, 29.1, 28.5, 26.0, 25.4, 22.8, 22.6, 18.6, 14.2. HRMS (+ESI-TOF) m/z for $C_{49}H_{75}N_5O_9$ $[M+Na]^+$ calculated: 776.3913, found: 776.3911

6-(12-Fluorododecanamido)-*N*-phenylhexanamide (8)

6-Amino-*N*-phenylhexanamide (AHA[1]) (0.10 g, 0.48 mmol) was dissolved in acetonitrile with 12-fluorododecanoic acid, a catalyst amount of DMAP (few crystals), and 1 equivalent of DCC (275 mg). The mixture was stirred under argon at 22°C for 12 hours. The solvent was evaporated and the residue was purified by column chromatography. The product was eluted with 3% MeOH in DCM and subsequently purified by semi-preparative HPLC to improve the purity, eluted as a single peak at 8 minutes with 50% acetonitrile/water. The final compound was isolated in 10% yield. ^{1}H NMR (400 MHz, DMSO-d_6) δ 9.85 (s, 1H), 7.72 (s, 1H), 7.56 (d, J = 7.85Hz, 2H), 7.25 (d, J = 1.8 Hz, 2H), 6.99 (t, J = 7.3 Hz, 1H), 4.44 (dt, J = 47.6 Hz, 2H), 2.99 (s, 2H), 2.25 (m, 2H), 1.98 (m, 2H), 1.54 (m, 4H), 1.37 (m, 4H), 1.20 (s, 10H).^{19}F NMR (376 MHz, DMSO-d_6) δ -216.36 – -216.88 (m). HRMS (+ESI-TOF) m/z for $C_{24}H_{40}FN_2O_2$ $[M+Na]^+$ calculated 407.3074, found 407.3083.

6-(12-Bromododecanamido)-*N*-phenylhexanamide (9)

DIPEA (0.13 mL, 0.73 mmol) was added to the mixture of 6-amino-*N*-phenylhexanamide (AHA) (prepared using previously published method[1]) (0.10 g, 0.48 mmol) and bromoundecanoic acid benzotriazole (0.21 g, 0.58 mmol) dissolved in DMF (5 mL), and left to stir at 22°C for 6 h. The solvent was then evaporated under reduced pressure; the crude was dissolved in DCM and washed with 2N hydrochloric acid. The organic layer was dried over sodium sulfate. Evaporation and purification with column chromatography (eluent - EtOAc) gave the desired precursor in 62% yield. ^{1}H NMR (400 MHz, $CDCl_3$) δ 8.25 (s, 1H), 7.55 (d, *J* = 7.9 Hz, 2H), 7.27 (t, *J* = 7.9 Hz, 2H), 7.06 (t, *J* = 7.4 Hz, 1H), 5.96 (t, *J* = 5.2 Hz, 1H), 3.38 (t, *J* = 6.9 Hz, 2H), 3.22 (dd, *J* = 13.2, 6.8 Hz, 2H), 2.35 (t, *J* = 7.4 Hz, 2H), 2.13 (t, *J* = 7.6 Hz, 2H), 1.88 – 1.77 (m, 2H), 1.76 – 1.66 (m, 2H), 1.61 – 1.45 (m, 4H), 1.43 – 1.31 (m, 4H), 1.24 (s, 10H). ^{13}C NMR (101 MHz, $CDCl_3$) δ 173.6, 171.8, 138.4, 128.9, 124.1, 119.9, 39.2, 37.3, 36.9, 34.2, 32.9, 29.6, 29.5, 29.5, 29.4, 29.4, 29.3,

28.8, 28.2, 26.4, 25.9, 25.0. HRMS (+ESI-TOF) m/z for $C_{24}H_{40}BrN_2O_2$ $[M+Na]^+$ calculated 467.2273, found 467.2283.

6-(12-Iodododecanamido)-*N*-phenylhexanamide (10)

6-(12-Bromododecanamido)-*N*-phenylhexanamide (100 mg) was dissolved in acetone and 4 equivalents of NaI was added at 22°C and the mixture was stirred for 80 hours. The solution was evaporated and purified with column chromatography (eluent – EtOAc) to give the desired product in 73% yield (80 mg). ^{1}H NMR (600 MHz, $CDCl_3$) δ 7.54 (d, *J* = 7.6 Hz, 3H), 7.31 (t, *J* = 7.8 Hz, 2H), 7.09 (t, *J* = 7.4 Hz, 1H), 5.63 (s, 1H), 3.26 (dd, *J* = 13.2, 6.7 Hz, 2H), 3.18 (t, *J* = 7.0 Hz, 2H), 2.37 (t, *J* = 7.3 Hz, 2H), 2.14 (t, *J* = 7.6, 2H), 1.85 – 1.72 (m, 4H), 1.63 – 1.57 (m, 2H), 1.57 – 1.51 (m, 2H), 1.44 – 1.34 (m, 4H), 1.26 (m, 12H). HRMS (+ESI-TOF) m/z for $C_{24}H_{40}IN_2O_2$ $[M+Na]^+$ calculated 515.2135, found 515.2131.

6-^{18}F-(12-fluorododecanamido)-*N*-phenylhexanamide (11)

The F-18 was obtained in kryptofix/K[^{18}F] complex in acetonitrile/water (1 mL) from the PET/Cyclotron Facility, Wayne State University (Detroit, MI) and transferred into a crimped 2 mL V-vial for azeotropic drying. Acetonitrile (0.4 mL) was added to the mixture, which was dried under a stream of argon at 90-100°C. A solution of **10** (2-3 mg) in dry acetonitrile (0.4 mL) was added to the dried kryptofix/ K[^{18}F] and heated to 85-90°C under argon with stirring for 20 minutes. After cooling, the reaction mixture was passed through a 900 mg Altech silica gel cartridge (Fisher Scientific, Waltham, MA) and eluted with 2 mL of 10% methanol in dichloromethane (DCM). The solvent was evaporated under a stream of argon to remove DCM and to reduce the volume to 0.5 mL. The **11** was purified by semipreparative HPLC (40% ACN in water) by collecting the peak at 9 min. The decay corrected radiochemical yield was 12% and the

compound purity was >95%, as assessed by analytical HPLC and co-elution with the non-radiolabeled reference standard **9**.

Additional Discussion Points:

Regarding the results of *in silico* modeling studies

Furthermore, results of current *in silico* modeling studies demonstrate that the distance between the carbonyl-carbon to the ADPR-nicotinamide ester linkage is smaller for longer chain leaving groups, such as the fluorododecanoyl derivatized Boc-Lys-AMC and AHA backbones. These *in silico* docking studies were conducted using currently available crystal structures of SIRT2, which depict the enzyme in either the “closed” or “open” confirmation (i.e. before or after enzymatic reaction with a substrate). Because there are no crystal structures available for SIRT2 in the transition state with bound NAD^+, we chose the structure of SIRT2 co-crystallized with SirReal2 (SIRT2 inhibitor) and NAD^+[9] (PDB: 4RMG). This crystal structure with full NAD^+ intact, rather than post cleavage with only ADPR present[10], is indicative of an enzyme in the open confirmation when a substrate initially docks inside the enzyme active site. This step is especially important for the SIRT enzymes, as the close proximity and accurate positioning between nicotinamide and the substrate carbonyl moiety is crucial for the reaction to occur. The root mean square deviation (r.m.s.d) for the central carbon during the transition from the “open” to “closed” conformation has been characterized in the literature[9], allowing estimation of the changes in key interaction distances. The r.m.s.d of the open versus closed configuration is about 1.7Å in PDB 4RMG. Using this correction factor, the distances between a leaving group carbonyl and a nicotinamide of NAD^+ ring fall into an acceptable range for reaction to occur. Other studies have explored the structural activity in 5 ns molecular dynamic simulations and noted similar findings for the structural changes in the SIRT2 active site[11]. Therefore, we hypothesize that the distance between the amide bond

carbonyl carbon and the nicotinamide cleavage site of NAD^+ plays an important role in substrate catalytic efficiency of SIRT enzymes. The mechanism of SIRT2 mediated cleavage of acylated lysine moieties involves simultaneous cleavage of nicotinamide and activation and subsequent cleavage of the acyl carbonyl carbon amide bond. Therefore, the selection of site for with substitution fluorine atom (strongly electronegative and electron withdrawing) is crucial for preservation of catalytic activity of SIRT2. For example, if a fluorine atom (or another electron withdrawing group) were to be placed in close proximity to the α-carbon, catalytic efficiency would decrease, as reported by us previously for fluro- and trifluoro-acetamidohexanoicanilide[12].

Regarding the role of SIRT2 in oncogenesis and progression

The role of SIRT2 in oncogenesis and progression of brain gliomas and other primary brain tumors is not yet well understood. Although some studies demonstrated downregulation of SIRT2 expression in about 70% of gliomas[13], it appears that the expression levels alone don't necessarily determine the enzymatic activity and mechanistic roles of SIRT2 in gliomagenesis, progression, and development of resistance to chemo- and radio- therapy. Furthermore, there is a controversy in the literature regarding the glioma promoting or suppressing roles of SIRT2. For example, one study[14] demonstrated that both genomic shRNA-mediated knockdown or pharmacologic inhibition (by AGK2) of SIRT2 expression-activity caused necrosis and caspase-3-dependent apoptosis of C6 rat glioma cells. In contrast, another study[15] demonstrated that in T98G, U87MG, and U251 cells the shRNA-mediated SIRT2 knockdown promoted colony formation, while adenoviral-mediated overexpression of SIRT2 repressed colony formation *in vitro*. The latter study has revealed the mechanism of SIRT2-mediated suppression of glioma cell growth occurred via negative regulation of miR-21 expression, which is known to be upregulated in gliomas and to promote glioma growth[16] and resistance to chemo-radiotherapy[17] . Therefore, additional studies

are required to determine whether the genetic knock-down or pharmacologic inhibition of SIRT2 expression in glioma cells *in vivo* will be therapeutically effective of will result in the accelerated growth and invasion. Such studies will be greatly facilitated by multi-modal molecular imaging with PET/CT/MRI and a SIRT2-specific radiotracer.

Regarding SIRT2 expression in various cancers

The development of a second-generation SIRT2-selective substrate-type radiotracer is very important because several recent studies demonstrated that the labeling index of nuclear-localized SIRT2 is significantly higher in glioblastomas (grade IV), as compared to astrocytomas (grade II) and normal brain tissue and strongly correlated with malignant progression and the overall survival of patients with glioblastomas[18]. The immunohistochemical staining of many tissues in the Human Protein Atlas displays intense staining of SIRT2 protein in all glioma tissues with greater magnitude than in breast or pancreatic cancer patient derived tissue samples (http://www.proteinatlas.org/search/sirt2). Higher levels of expression and higher labeling index for SIRT2 is also associated with progression and poor prognosis in patients with non-small cell lung cancer (NSCLC)[19] and cervical carcinoma[20]. Breast carcinomas with increased levels of SIRT2 expression have poorer prognosis as compared to those with lower expression levels[18]. Some other tumor types (i.e., melanomas), may harbor mutations in SIRT2 gene resulting in reduction of enzymatic activity by 80–90% compared to the wild-type protein, however consequences to tumor progression and overall prognosis are yet unknown[21]. Therefore, molecular imaging with PET using SIRT2-specific radiotracers should provide information about the location and magnitude of SIRT2 expression and activity in tumors non-invasively and in real time. This may help to determine the mechanistic, therapeutic, and prognostic roles of SIRT2 in different cancers as well as monitoring novel SIRT2 targeted therapies.

1. Mukhopadhyay, U.; Tong, W. P.; Gelovani, J. G.; Alauddin, M. M., Radiosynthesis of 6-([18F]fluoroacetamido)-1-hexanoicanilide ([18F]FAHA) for PET imaging of histone deacetylase (HDAC). *Journal of Labelled Compounds and Radiopharmaceuticals* **2006,** *49* (11), 997-1006.
2. Mukhopadhyay U, T. W., **Gelovani J**, Alauddin MM, Radiosynthesis of 6-(^{18}F-fluoroacetamido)-1-hexanoicanilide (^{18}F-FAHA) for PET Imaging of histone deacetylase (HDAC). *J Labelled Comp Radiopharm* **2006,** *49*, 997-1006.
3. (a) Marcotte, P. A.; Richardson, P. R.; Guo, J.; Barrett, L. W.; Xu, N.; Gunasekera, A.; Glaser, K. B., Fluorescence assay of SIRT protein deacetylases using an acetylated peptide substrate and a secondary trypsin reaction. *Analytical biochemistry* **2004,** *332* (1), 90-99; (b) Huhtiniemi, T.; Salo, H. S.; Suuronen, T.; Poso, A.; Salminen, A.; Leppanen, J.; Jarho, E.; Lahtela-Kakkonen, M., Structure-based design of pseudopeptidic inhibitors for SIRT1 and SIRT2. *J Med Chem* **2011,** *54* (19), 6456-68.
4. Zhu, K.; Borrelli, K. W.; Greenwood, J. R.; Day, T.; Abel, R.; Farid, R. S.; Harder, E., Docking Covalent Inhibitors: A Parameter Free Approach To Pose Prediction and Scoring. *Journal of Chemical Information and Modeling* **2014,** *54* (7), 1932-1940.
5. Sastry, G. M.; Adzhigirey, M.; Day, T.; Annabhimoju, R.; Sherman, W., Protein and ligand preparation: parameters, protocols, and influence on virtual screening enrichments. *J Comput Aided Mol Des* **2013,** *27* (3), 221-234.
6. Shelley, J. C.; Cholleti, A.; Frye, L. L.; Greenwood, J. R.; Timlin, M. R.; Uchimaya, M., Epik: a software program for pK a prediction and protonation state generation for drug-like molecules. *J Comput Aided Mol Des* **2007,** *21* (12), 681-691.
7. Friesner, R. A.; Banks, J. L.; Murphy, R. B.; Halgren, T. A.; Klicic, J. J.; Mainz, D. T.; Repasky, M. P.; Knoll, E. H.; Shelley, M.; Perry, J. K.; Shaw, D. E.; Francis, P.; Shenkin, P. S., Glide: A New Approach for Rapid, Accurate Docking and Scoring. 1. Method and Assessment of Docking Accuracy. *Journal of Medicinal Chemistry* **2004,** *47* (7), 1739-1749.
8. Yeh, S. H.-H.; Lin, C.-F.; Kong, F.-L.; Wang, H.-E.; Hsieh, Y.-J.; Gelovani, J. G.; Liu, R.-S., Molecular Imaging of Nonsmall Cell Lung Carcinomas Expressing Active Mutant EGFR Kinase Using PET with [(124)I]-Morpholino-IPQA. *BioMed Research International* **2013,** *2013*, 549359.
9. Rumpf, T.; Schiedel, M.; Karaman, B.; Roessler, C.; North, B. J.; Lehotzky, A.; Oláh, J.; Ladwein, K. I.; Schmidtkunz, K.; Gajer, M.; Pannek, M.; Steegborn, C.; Sinclair, D. A.; Gerhardt, S.; Ovádi, J.; Schutkowski, M.; Sippl, W.; Einsle, O.; Jung, M., Selective Sirt2 inhibition by ligand-induced rearrangement of the active site. *Nat Commun* **2015,** *6*.
10. Moniot, S.; Steegborn, C.; Schutkowski, M., Crystal structure analysis of human Sirt2 and its ADP-ribose complex. *Journal of Structural Biology* **2013,** *182* (2), 136-143.
11. Sakkiah, S.; Arooj, M.; Cao, G. P.; Lee, K. W., Insight the C-Site Pocket Conformational Changes Responsible for Sirtuin 2 Activity Using Molecular Dynamics Simulations. *PloS one* **2013,** *8* (3), e59278.
12. (a) Bonomi, R.; Mukhopadhyay, U.; Shavrin, A.; Yeh, H.-H.; Majhi, A.; Dewage, S. W.; Najjar, A.; Lu, X.; Cisneros, G. A.; Tong, W. P.; Alauddin, M. M.; Liu, R.-S.; Mangner, T. J.; Turkman, N.; Gelovani, J. G., Novel Histone Deacetylase Class IIa Selective Substrate Radiotracers for PET Imaging of Epigenetic Regulation in the Brain. *PloS one* **2015,** *10* (8), e0133512; (b) Yeh, H.-H.; Tian, M.; Hinz, R.; Young, D.; Shavrin, A.; Mukhapadhyay, U.; Flores, L. G.; Balatoni, J.; Soghomonyan, S.; Jeong, H. J.; Pal, A.; Uthamanthil, R.; Jackson, J. N.; Nishii, R.; Mizuma, H.; Onoe, H.; Kagawa, S.; Higashi, T.; Fukumitsu, N.; Alauddin, M.; Tong, W.; Herholz, K.; Gelovani, J. G., Imaging epigenetic regulation by histone deacetylases in the brain using PET/MRI with 18F-FAHA. *NeuroImage* **2013,** *64* (0), 630-639.

13. Inoue, T.; Nakayama, Y.; Yamada, H.; Li, Y. C.; Yamaguchi, S.; Osaki, M.; Kurimasa, A.; Hiratsuka, M.; Katoh, M.; Oshimura, M., SIRT2 downregulation confers resistance to microtubule inhibitors by prolonging chronic mitotic arrest. *Cell Cycle* **2009,** *8* (8), 1279-91.
14. He, X.; Nie, H.; Hong, Y.; Sheng, C.; Xia, W.; Ying, W., SIRT2 activity is required for the survival of C6 glioma cells. *Biochemical and biophysical research communications* **2012,** *417* (1), 468-72.
15. Li, Y.; Dai, D.; Lu, Q.; Fei, M.; Li, M.; Wu, X., Sirt2 suppresses glioma cell growth through targeting NF-kappaB-miR-21 axis. *Biochemical and biophysical research communications* **2013,** *441* (3), 661-7.
16. Chan, J. A.; Krichevsky, A. M.; Kosik, K. S., MicroRNA-21 is an antiapoptotic factor in human glioblastoma cells. *Cancer research* **2005,** *65* (14), 6029-33.
17. Shi, L.; Chen, J.; Yang, J.; Pan, T.; Zhang, S.; Wang, Z., MiR-21 protected human glioblastoma U87MG cells from chemotherapeutic drug temozolomide induced apoptosis by decreasing Bax/Bcl-2 ratio and caspase-3 activity. *Brain research* **2010,** *1352*, 255-64.
18. Imaoka, N.; Hiratsuka, M.; Osaki, M.; Kamitani, H.; Kambe, A.; Fukuoka, J.; Kurimoto, M.; Nagai, S.; Okada, F.; Watanabe, T.; Ohama, E.; Kato, S.; Oshimura, M., Prognostic significance of sirtuin 2 protein nuclear localization in glioma: an immunohistochemical study. *Oncology reports* **2012,** *28* (3), 923-30.
19. Grbesa, I.; Pajares, M. J.; Martinez-Terroba, E.; Agorreta, J.; Mikecin, A. M.; Larrayoz, M.; Idoate, M. A.; Gall-Troselj, K.; Pio, R.; Montuenga, L. M., Expression of sirtuin 1 and 2 is associated with poor prognosis in non-small cell lung cancer patients. *PloS one* **2015,** *10* (4), e0124670.
20. Singh, S.; Kumar, P. U.; Thakur, S.; Kiran, S.; Sen, B.; Sharma, S.; Rao, V. V.; Poongothai, A. R.; Ramakrishna, G., Expression/localization patterns of sirtuins (SIRT1, SIRT2, and SIRT7) during progression of cervical cancer and effects of sirtuin inhibitors on growth of cervical cancer cells. *Tumour biology : the journal of the International Society for Oncodevelopmental Biology and Medicine* **2015,** *36* (8), 6159-71.
21. Lennerz, V.; Fatho, M.; Gentilini, C.; Frye, R. A.; Lifke, A.; Ferel, D.; Wolfel, C.; Huber, C.; Wolfel, T., The response of autologous T cells to a human melanoma is dominated by mutated neoantigens. *Proc Natl Acad Sci U S A* **2005,** *102* (44), 16013-8.

Supplementary Figures

A novel substrate radiotracer for molecular imaging of SIRT2 expression and activity with positron emission tomography.

Robin E. Bonomi[1], Maxwell Laws[1], Vadim Popov[1], Swatabdi Kamal[1], Shreya Potukutchi[1], Shavrin[1], Xin Lu[2], Nashaat Turkman[1], Ren Shuan Liu[3], Thomas Mangner[2], Juri G. Gelovani[1]*

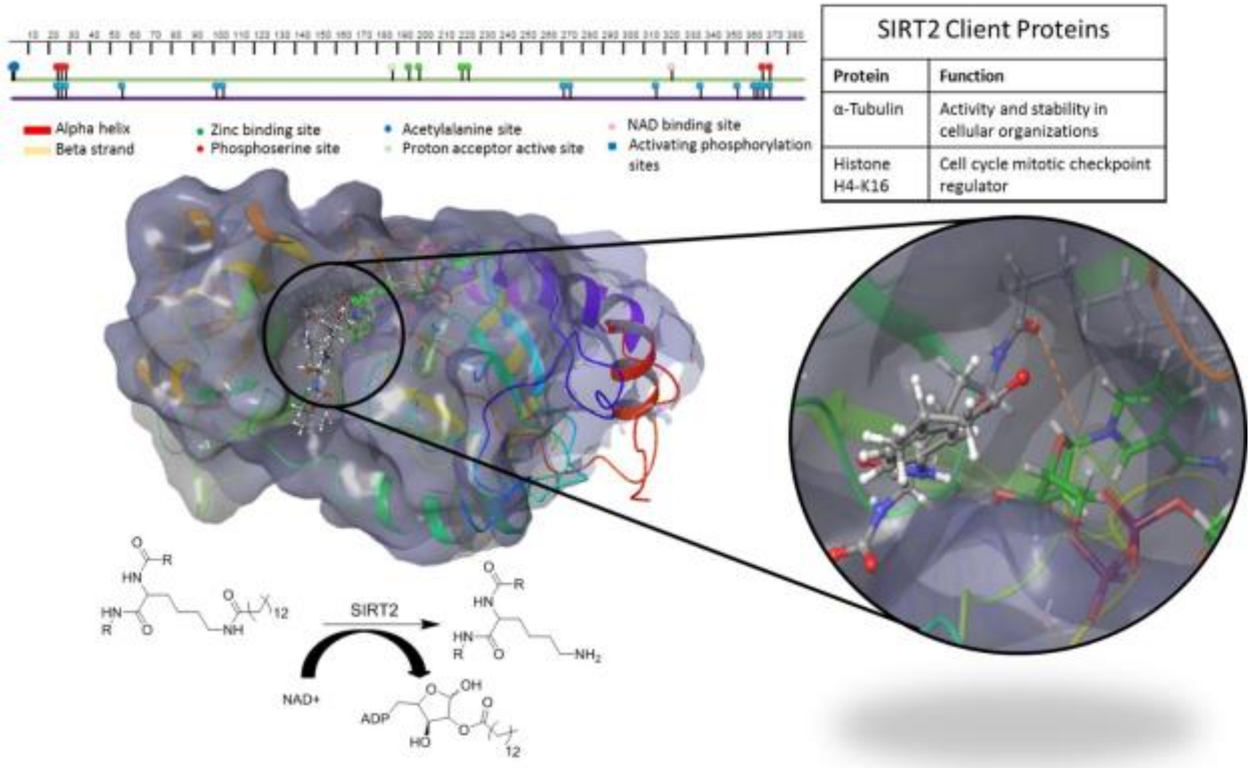

Fig. S1. The structure of SIRT2 docked with the Boc-Lys(12F-dodecanoyl)-AMC and NAD^+ adapted from crystal structure 4RMG. The SIRT2 sites of phosphorylation and binding highlighted on the residue chart. The SIRT2 client proteins are also listed in the table and the mechanism for NAD-dependent SIRT2 mediated cleavage of long acyl chains is depicted here. The process of SIRT2-mediated cleavage and leaving group entrapment is depicted within the cellular representation.

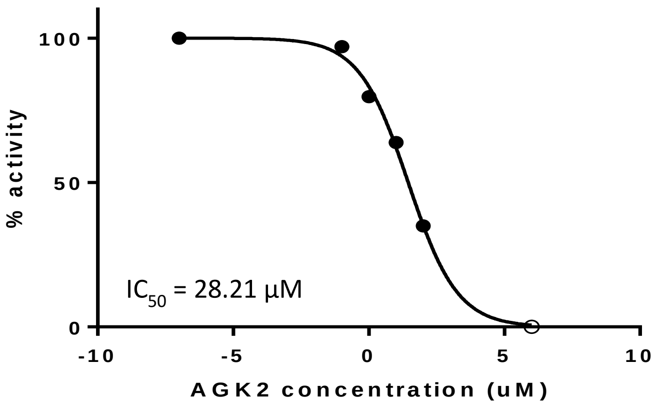

Fig. S2. Boc-Lys(12F-dodecanoyl)-AMC (**4**) was used to experimentally determine the IC_{50} value for a known inhibitor AGK2.

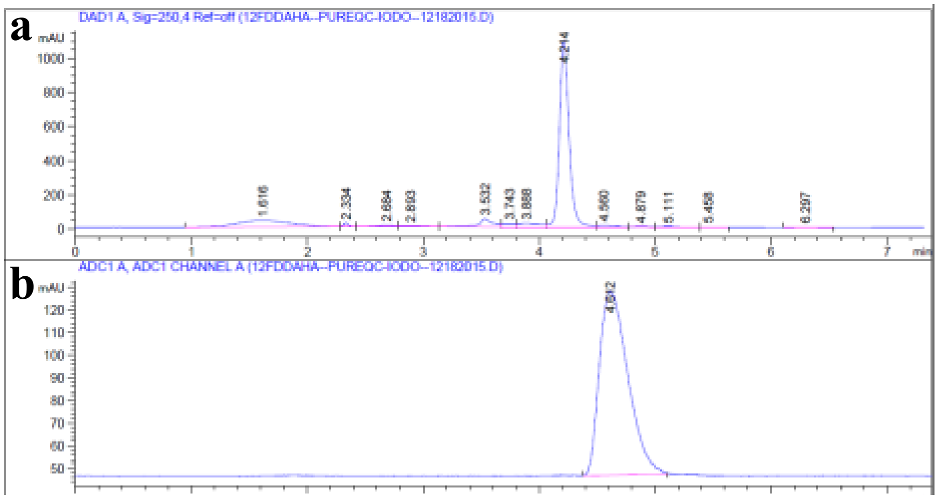

Fig. S3. The quality control chromatogram of the 12-[^{18}F]DDAHA (**11**) radiotracer as analyzed by HPLC demonstrates high radiochemical purity and very small amount of UV impurities. **A)** The UV non-radiolabeled standard chromatogram (**10**) **B)** The radio-HPLC chromatogram for the labeled 12-[18F]DDAHA, (**11**).

a

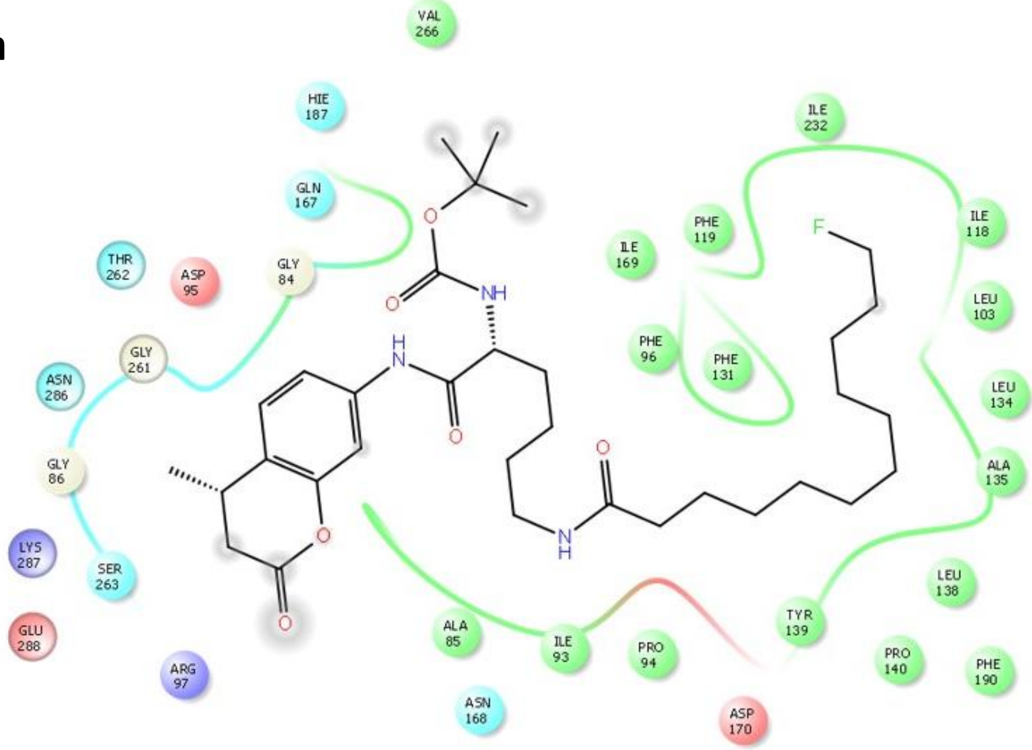

b

Compound Name	MW (g/mol)	Docking Score	Distance ADP–carbonyl (Å)	Distance after closing (Å)
Boc-lys(12F)-AMC	619	-8.925	4.30	3.6
Boc-lys(myr)-AMC	630	-7.977	5.52	3.8
Boc-lys(10F)-AMC	592	-9.304	5.50	3.8
Boc-lys(6F)-AMC	522	-8.524	5.97	4.2
12-DDFAHA	405	-9.070	7.22	5.5

Fig.S4 A) The 2D representation of the interactions between SIRT2 and the substrate, Boc-Lys(12F-dodecanyl)-AMC. The 12-fluoro dodecanyl leaving group has similar interactions to those in literature, evidence that the leaving group is fitting in the correct position. **B)** The table of results from our compound docking study. The compound name, MW, docking score (where more negative is a better fit), and most importantly, the distance between the 1' postion of the ADP ring and the carbonyl of the leaving group. This distance is also shown after the 1.7Å correction for catalytic conformational change.
